# Burden of infectious disease studies in Europe and the United Kingdom: a review of methodological design choices

**DOI:** 10.1017/S0950268823000031

**Published:** 2023-01-09

**Authors:** Periklis Charalampous, Juanita A. Haagsma, Lea S. Jakobsen, Vanessa Gorasso, Isabel Noguer, Alicia Padron-Monedero, Rodrigo Sarmiento, João Vasco Santos, Scott A. McDonald, Dietrich Plass, Grant M. A. Wyper, Ricardo Assunção, Elena von der Lippe, Balázs Ádám, Ala'a AlKerwi, Jalal Arabloo, Ana Lúcia Baltazar, Boris Bikbov, Maria Borrell-Pages, Iris Brus, Genc Burazeri, Serafeim C. Chaintoutis, José Chen-Xu, Nino Chkhaberidze, Seila Cilovic-Lagarija, Barbara Corso, Sarah Cuschieri, Carlotta Di Bari, Keren Dopelt, Mary Economou, Theophilus I. Emeto, Peter Fantke, Florian Fischer, Alberto Freitas, Juan Manuel García-González, Federica Gazzelloni, Mika Gissler, Artemis Gkitakou, Hakan Gulmez, Sezgin Gunes, Sebastian Haller, Romana Haneef, Cesar A. Hincapié, Paul Hynds, Jane Idavain, Milena Ilic, Irena Ilic, Gaetano Isola, Zubair Kabir, Maria Kamusheva, Pavel Kolkhir, Naime Meriç Konar, Polychronis Kostoulas, Mukhtar Kulimbet, Carlo La Vecchia, Paolo Lauriola, Miriam Levi, Marjeta Majer, Enkeleint A. Mechili, Lorenzo Monasta, Stefania Mondello, Javier Muñoz Laguna, Evangelia Nena, Edmond S. W. Ng, Paul Nguewa, Vikram Niranjan, Iskra Alexandra Nola, Rónán O'Caoimh, Marija Obradović, Elena Pallari, Mariana Peyroteo, Vera Pinheiro, Nurka Pranjic, Miguel Reina Ortiz, Silvia Riva, Cornelia Melinda Adi Santoso, Milena Santric Milicevic, Tugce Schmitt, Niko Speybroeck, Maximilian Sprügel, Paschalis Steiropoulos, Aleksandar Stevanovic, Lau Caspar Thygesen, Fimka Tozija, Brigid Unim, Hilal Bektaş Uysal, Orsolya Varga, Milena Vasic, Rafael José Vieira, Vahit Yigit, Brecht Devleesschauwer, Sara M. Pires

**Affiliations:** 1 Department of Public Health, Erasmus MC University Medical Center, Rotterdam, The Netherlands; 2 National Food Institute, Technical University of Denmark, Lyngby, Denmark; 3 Department of Public Health and Primary Care, Ghent University, Ghent, Belgium; 4 Department of Epidemiology and Public Health, Sciensano, Brussels, Belgium; 5 National School of Public Health, Carlos III Institute of Health, Madrid, Spain; 6 Medicine School, University of Applied and Environmental Sciences, Bogota, Colombia; 7 CINTESIS@RISE – Centre for Health Technology and Services Research, Health Research Network, Faculty of Medicine of the University of Porto, Porto, Portugal; 8 MEDCIDS – Department of Community Medicine, Information and Health Decision Sciences, Faculty of Medicine, University of Porto, Porto, Portugal; 9 Public Health Unit, Agrupamentos de Centro de Saúde Grande Porto V – Porto Ocidental, Administração Regional de Saúde do Norte, Porto, Portugal; 10 Centre for Infectious Disease Control, National Institute for Public Health and the Environment (RIVM), Bilthoven, The Netherlands; 11 Department for Exposure Assessment and Environmental Health Indicators, German Environment Agency, Berlin, Germany; 12 School of Health & Wellbeing, University of Glasgow, Glasgow, UK; 13 Food and Nutrition Department, National Institute of Health Dr. Ricardo Jorge, Lisbon, Portugal; 14 Department of Epidemiology and Health Monitoring, Robert Koch Institute, Berlin, Germany; 15 Institute of Public Health, College of Medicine and Health Sciences, United Arab Emirates University, Al Ain, United Arab Emirates; 16 Directorate of Health, Service Epidemiology and Statistics, Luxembourg, Luxembourg; 17 Health Management and Economics Research Center, Health Management Research Institute, Iran University of Medical Sciences, Tehran, Iran; 18 Scientific-Pedagogical Unit of Dietetics and Nutrition, Coimbra Health School, Polytechnic Institute of Coimbra, Coimbra, Portugal; 19 Dipartimento di Politiche per la Salute, Istituto di Ricerche Farmacologiche Mario Negri IRCCS, Milan, Italy; 20 Cardiovascular Program ICCC, Institut d'Investigació Biomèdica Sant Pau (IIB-Sant Pau), Barcelona, Spain; 21 Department of Public Health, Faculty of Medicine, University of Medicine, Tirana, Albania; 22 Diagnostic Laboratory, School of Veterinary Medicine, Faculty of Health Sciences, Aristotle University of Thessaloniki, Thessaloniki, Greece; 23 NOVA National School of Public Health, Public Health Research Centre, Universidade NOVA de Lisboa, Lisbon, Portugal; 24 Public Health Unit, Primary Health Care Cluster Baixo Mondego, Coimbra, Portugal; 25 Department of Medical Statistics, National Center for Disease Control and Public Health of Georgia, Georgia, Georgia; 26 Institute for Public Health FB&H, Sarajevo, Bosnia and Herzegovina; 27 Neuroscience Institute, National Research Council, Padova, Italy; 28 Department of Anatomy, Faculty of Medicine and Surgery, University of Malta, Msida, Malta; 29 Department of Public Health, Ashkelon Academic College, Ashkelon, Israel; 30 Department of Health Policy and Management, School of Public Health, Faculty of Health Sciences, Ben-Gurion University of the Negev, Beer Sheva, Israel; 31 Department of Nursing, School of Health Sciences, Cyprus University of Technology, Limassol, Cyprus; 32 Public Health & Tropical Medicine, College of Public Health, Medical and Veterinary Sciences, James Cook University, Townsville, Australia; 33 World Health Organization Collaborating Center for Vector-Borne and Neglected Tropical Diseases, College of Public Health, Medical and Veterinary Sciences, James Cook University, Townsville, Australia; 34 Australian Institute of Tropical Health and Medicine, James Cook University, Townsville, Australia; 35 Quantitative Sustainability Assessment, Department of Environmental and Resource Engineering, Technical University of Denmark, Lyngby, Denmark; 36 Institute of Public Health, Charité – Universitätsmedizin Berlin, Berlin, Germany; 37 Department of Sociology, Universidad Pablo de Olavide, Seville, Spain; 38 Institute and Faculty of Actuaries, London, UK; 39 Department of Knowledge Brokers, Finnish Institute for Health and Welfare (THL), Helsinki, Finland; 40 Department of Molecular Medicine and Surgery, Karolinska Institute, Stockholm, Sweden; 41 Academic Primary Health Care Centre, Stockholm, Sweden; 42 Research Centre for Child Psychiatry and Invest Research Flagship, University of Turku, Turku, Finland; 43 Department of Internal Medicine and Epidemiology, Erasmus MC University Medical Center, Rotterdam, The Netherlands; 44 Department of Family Medicine, Faculty of Medicine, İzmir Democracy University, Izmir, Turkey; 45 Department of Multidisciplinary Molecular Medicine, Graduate Institute, Ondokuz Mayis University, Samsun, Turkey; 46 Department of Medical Biology, Medical Faculty, Ondokuz Mayis University, Samsun, Turkey; 47 Department of Infectious Disease Epidemiology, Robert Koch Institute, Berlin, Germany; 48 Department of Non-Communicable Diseases and Injuries, Santé Publique France, Saint-Maurice, France; 49 EBPI-UZWH Musculoskeletal Epidemiology Research Group, University of Zurich and Balgrist University Hospital, Zurich, Switzerland; 50 Epidemiology, Biostatistics and Prevention Institute (EBPI), University of Zurich, Zurich, Switzerland; 51 University Spine Centre Zurich (UWZH), Balgrist University Hospital, University of Zurich, Zurich, Switzerland; 52 Environmental Sustainability and Health Institute, Technological University Dublin, Dublin, Ireland; 53 Department of Health Statistics, National Institute for Health Development, Tallinn, Estonia; 54 Department of Epidemiology, Faculty of Medical Sciences, University of Kragujevac, Kragujevac, Serbia; 55 Faculty of Medicine, University of Belgrade, Belgrade, Serbia; 56 Department of General Surgery and Surgical-Medical Specialties, School of Dentistry, University of Catania, Catania, Italy; 57 Public Health & Epidemiology, School of Public Health, University College Cork, Cork, Ireland; 58 Department of Organization & Economics of Pharmacy, Faculty of Pharmacy, Medical University of Sofia, Sofia, Bulgaria; 59 Institute of Allergology, Charité – Universitätsmedizin Berlin, Freie Universität Berlin, and Humboldt-Universität zu Berlin, Berlin, Germany; 60 Fraunhofer Institute for Translational Medicine and Pharmacology ITMP, Allergology and Immunology, Berlin, Germany; 61 Department of Biostatistics and Medical Informatics, Faculty of Medicine, Kirsehir Ahi Evran University, Kirsehir, Turkey; 62 Laboratory of Epidemiology and Artificial Intelligence, Faculty of Public Health, University of Thessaly, Thessaly, Greece; 63 Health Research Institute, Al Farabi Kazakh National University, Almaty, Kazakhstan; 64 Atchabarov Scientific Research Institute of Fundamental Medicine, Asfendiyarov Kazakh National Medical University, Almaty, Kazakhstan; 65 Department of Clinical Sciences and Community Health, University of Milan, Milan, Italy; 66 Italian Network of Sentinel Physicians for the Environment (RIMSA), International Society Doctors for the Environment (ISDE), Federazione Nazione Ordine dei Medici (FNOMCeO), Arezzo, Italy; 67 Epidemiology Unit, Department of Prevention, Local Health Authority Tuscany Centre, Florence, Italy; 68 Andrija Štampar School of Public Health, School of Medicine, University of Zagreb, Zagreb, Croatia; 69 Clinic of Social and Family Medicine, School of Medicine, University of Crete, Crete, Greece; 70 Department of Healthcare, Faculty of Public Health, University of Vlora, Vlora, Albania; 71 Institute of Maternal, Child Health – IRCCS Burlo Garofolo, Trieste, Italy; 72 Department of Biomedical and Dental Sciences and Morphofunctional Imaging, University of Messina, Messina, Italy; 73 Laboratory of Social Medicine, Medical School, Democritus University of Thrace, Alexandroupolis, Greece; 74 Faculty of Epidemiology and Population Health, London School of Hygiene & Tropical Medicine, London, UK; 75 Department of Microbiology and Parasitology, ISTUN Institute of Tropical Health, IdiSNA (Navarra Institute for Health Research), University of Navarra, Pamplona, Spain; 76 School of Public Health, Physiotherapy and Sport Sciences, University College Dublin, Dublin, Ireland; 77 Department of Geriatric Medicine, Mercy University Hospital, Grenville Place, Cork City, Ireland; 78 Department of Gerontology and Rehabilitation, University College Cork, St Finbarr's Hospital, Douglas road, Cork City, Ireland; 79 Department of Preventive and Pediatric Dentistry, Faculty of Medicine, University of Banja Luka, Bosnia, Herzegovina; 80 Health Innovation Network, Minerva House, Montague Cl, London, UK; 81 Comprehensive Health Research Centre, NOVA Medical School, Universidade NOVA de Lisboa, Lisboa, Portugal; 82 Public Health Unit, Matosinhos Local Health Unit, Matosinhos, Portugal; 83 CINTESIS – Centre for Health Technology and Services Research, Faculty of Medicine of the University of Porto, Porto, Portugal; 84 Department of Occupational Medicine, School of Medicine, University of Tuzla, Tuzla, Bosnia and Herzegovina; 85 School of Public and Population Health, Boise State University, Boise, USA; 86 Department of Psychology and Pedagogic Science, St Mary's University, London, UK; 87 Faculty of Public Health, University of Debrecen, Debrecen, Hungary; 88 Institute of Social Medicine, Faculty of Medicine, University of Belgrade, Belgrade, Serbia; 89 Department of International Health, Care and Public Health Research Institute – CAPHRI, Faculty of Health, Medicine and Life Sciences, Maastricht University, Maastricht, The Netherlands; 90 Institute of Health and Society (IRSS), Université catholique de Louvain, Brussels, Belgium; 91 Department of Neurology, Friedrich-Alexander-Universität Erlangen-Nürnberg, Erlangen, Germany; 92 Department of Respiratory Medicine, Medical School, Democritus University of Thrace, Alexandroupolis, Greece; 93 National Institute of Public Health, University of Southern Denmark, Copenhagen, Denmark; 94 Faculty of Medicine, Saints Cyril and Methodius University of Skopje, Skopje, North Macedonia; 95 Department of Cardiovascular, Endocrine-Metabolic Diseases and Aging, Istituto Superiore Di Sanità, Rome, Italy; 96 Department of Internal Medicine, Adnan Menderes University School of Medicine, Aydin, Turkey; 97 Department of Public Health and Epidemiology, Faculty of Medicine, University of Debrecen, Debrecen, Hungary; 98 Faculty of Dentistry Pancevo, University Business Academy in Novi Sad, Pancevo, Serbia; 99 Institute of Public Health of Serbia Dr Milan Jovanović Batut, Belgrade, Serbia; 100 Department of Health Management, Suleyman Demirel University, Isparta, Turkey; 101 Department of Translational Physiology, Infectiology and Public Health, Ghent University, Merelbeke, Belgium

**Keywords:** Burden of disease, disability-adjusted life years, infectious diseases, methodology, systematic review

## Abstract

This systematic literature review aimed to provide an overview of the characteristics and methods used in studies applying the disability-adjusted life years (DALY) concept for infectious diseases within European Union (EU)/European Economic Area (EEA)/European Free Trade Association (EFTA) countries and the United Kingdom. Electronic databases and grey literature were searched for articles reporting the assessment of DALY and its components. We considered studies in which researchers performed DALY calculations using primary epidemiological data input sources. We screened 3053 studies of which 2948 were excluded and 105 studies met our inclusion criteria. Of these studies, 22 were multi-country and 83 were single-country studies, of which 46 were from the Netherlands. Food- and water-borne diseases were the most frequently studied infectious diseases. Between 2015 and 2022, the number of burden of infectious disease studies was 1.6 times higher compared to that published between 2000 and 2014. Almost all studies (97%) estimated DALYs based on the incidence- and pathogen-based approach and without social weighting functions; however, there was less methodological consensus with regards to the disability weights and life tables that were applied. The number of burden of infectious disease studies undertaken across Europe has increased over time. Development and use of guidelines will promote performing burden of infectious disease studies and facilitate comparability of the results.

## Introduction

Despite substantial progress in the prevention and treatment of infectious diseases, it is evident that the health impact of infectious diseases is still considerable worldwide. While the population health impact of some infectious diseases has decreased, new infectious diseases have emerged, such as the coronavirus disease 2019 (COVID-19), and there has also been an upsurge in other infectious diseases, such as the enterovirus D68 infections and scarlet fever [1–4]. In Europe, several factors have contributed to the altered landscape of many (re-)emerging infectious diseases with a significant impact on populations' health, including demographic changes such as population ageing, fertility, migration, zoonotic spillover events and environmental changes including climate change [5]. The changes in the burden of infectious diseases call for population health metrics that allow for ranking and prioritisation between pathogens and guidance of surveillance systems.

Traditionally, the population health impact of infectious diseases has been quantified by the number of deaths and (lab) confirmed incident or prevalent cases attributable to a specific pathogen [6–8]. However, the heterogeneity of the clinical course and mortality rates of infectious diseases and possible long-term disabilities resulting from infections underlines the importance of considering mortality and morbidity simultaneously when assessing and comparing the impact of infectious diseases on population health. A prominent metric of population health that integrates mortality and morbidity is the disability-adjusted life year (DALY) [9]. This composite metric quantifies the health losses, by summing premature mortality, expressed in years of life lost due to premature mortality (YLL) and morbidity, expressed in years lived with disability (YLD) [10, 11].

To date, there have been three large-scale multi-country studies using the DALY metric to assess the impact of infectious diseases, namely the Burden of Communicable Diseases in Europe (BCoDE) [12], the Global Burden of Disease (GBD) study [13] and the World Health Organization (WHO) Estimates of the Global Burden of Food-borne Diseases [14]. The BCoDE study aimed to provide estimates of the current and future burden of infectious diseases in the European Union (EU) member states and European Economic Area/European Free Trade Association (EEA/EFTA) countries and the United Kingdom [12]. The GBD study includes estimations of incidence, prevalence, mortality, YLL, YLD and DALY for numerous infectious diseases and aims to provide information on the disease burden trends across 204 countries and territories, by age, sex and year for 1990–2019 [13]. Moreover, the WHO Food-borne Disease Burden Epidemiology Reference Group (WHO/FERG) studies aimed to estimate the global burden of food-borne diseases using epidemiological information gathered by over 30 hazards [14, 15].

However, the GBD, BCoDE and WHO/FERG studies have used different methodological approaches to estimate DALYs for infectious diseases. A major methodological choice relating to the YLD calculations of infectious disease is whether to use a prevalence- or incidence-based approach [16, 17]. The GBD study employs a prevalence-based approach which captures the current state of population health, by taking the prevalent cases at a specific point of time [13]. In contrast, the BCoDE and WHO/FERG studies employ an incidence-based approach, capturing the current and projected future burden of infections by taking the newly diagnosed cases and the average duration until recovery or death [18, 19]. Furthermore, burden of infectious disease studies require a choice between an outcome- or pathogen-based approach. The GBD study follows an outcome-based approach which assigns the disease burden to clinically defined categories of health conditions and provides estimates of the burden of disease of major infectious disease-related outcomes, such as diarrhoea or lower respiratory infections, for aetiologies. For instance, the GBD study includes *Campylobacter* spp., as aetiology for diarrhoea, limiting the associated health states to diarrhoea [13]. In contrast, the BCoDE and WHO/FERG studies follow a pathogen-based approach that aimed to capture major outcomes attributable to a specific pathogen, including sequelae [15, 18–21]. Another methodological choice relating to the YLD calculations is the set of disability weights that is applied to infectious-related health states. A disability weight reflects the relative severity of a health state with a value anchored between 0 (equivalent to full health) and 1 (equivalent to death). Several sets of disability weights are available, each reflecting different elicitation techniques [22, 23]. The GBD study applies the GBD 2013 set of disability weights [24], the BCoDE study applies the disability weights set for Europe [25], whereas the WHO/FERG studies used disability weights from the GBD 2010 and/or GBD 2013 studies [24]. Other methodological differences include different choice of life tables [17] and the use of social weighting functions, namely age-weighting (i.e. implies that value of life depends on age; a greater weight is assigned to deaths at younger ages and a lower weight to deaths at older ones) and time-discounting (i.e. implies a greater amount of DALYs when interventions apply in the present than in the future; this choice is mostly used for economic evaluations) [26]. To calculate YLL, the value of age-conditional life expectancy is required and is usually yielded from national or aspirational life tables. The GBD, BCoDE and WHO/FERG studies use aspirational life tables which are unisex, abridged and allow for internationally comparable results [13, 18, 19]. Social value choices were not applied in BCoDE and WHO/FERG estimations, and from 2010 onwards, these have been dropped in GBD study estimates.

Parallel to these multi-country studies, many independent burden of infectious disease studies (i.e. studies for which researchers performed own YLL, YLD and/or DALY calculations using primary epidemiological input sources) have been conducted across Europe over the years. These studies have varied in terms of scope and methodologies applied. Previous systematic reviews of burden of disease studies, focused on non-communicable diseases and injuries, have revealed considerable variations in methodological approaches used in independent disease burden studies [27–31]. Insight into these variations in methods is important since it affects the calculation, interpretation and comparability. Furthermore, an overview of methodological approaches of independent burden of infectious disease studies is currently lacking.

This systematic literature review aimed to provide an overview of the characteristics and methodologies that have been used in independent burden of infectious disease studies applying the DALY concept within EU/EEA/EFTA countries and the United Kingdom in order to identify methodological differences and provide future recommendations for conducting burden of infectious disease studies. The following key questions were addressed:

- In which countries have independent burden of infectious disease studies been performed?
- For which infectious diseases have independent burden of infectious disease studies been performed?
- Which methodological approaches have been used to assess mortality and morbidity in these independent studies?

## Methods

This review was part of a series of systematic literature reviews undertaken by the European Burden of Disease Network [32]. The burden-eu network aims to address challenges in disease burden estimates by identifying and comparing methods used in, and approaches towards, existing burden of disease studies. This review adheres to the Preferred Reporting Items for Systematic Reviews and Meta-analyses (PRISMA) Statement [33]. A protocol was registered on PROSPERO under ID CRD42020177477.

### Data sources and search strategy

With the assistance of a specialist librarian from the Erasmus MC, we searched for burden of disease studies using five electronic bibliographic databases, platforms and search engines in week 22, 2022. We also performed a manual search for grey literature via public health websites from all the EU/EEA/EFTA countries to retrieve governmental documents. To foster comprehensiveness in grey literature searches, we asked the burden-eu members to provide eligible grey literature from their countries. We also searched for grey literature via other websites which are included in the Supplementary Material. Finally, we checked the reference lists of eligible systematic reviews identified in the above searches. Details of the search strategy and list of the targeted EU/EEA/EFTA countries and the United Kingdom and public health websites can be found in the Supplementary Material.

### Inclusion and exclusion criteria

We restricted our analysis to studies assessing the burden due to infectious diseases in terms of YLL, YLD and/or DALY, utilizing their own national or sub-national data based on primary data input sources. We included multi-national studies, as long as they fulfilled the criteria above. Since the DALY concept was introduced in the ‘*World Development Report 1993: Investing in Health*’ [34], we considered only disease burden studies published after January 1990. We considered studies in which the infection was defined as an illness due to a pathogen arising through transmission from an infected individual (i.e. human–human transmission), an infected animal (i.e. direct animal contact) or from other pathways (i.e. food, travel, etc.) [35].

We excluded studies that performed secondary or systematic analyses based on the GBD estimates. We also excluded studies that did not assess the direct or indirect impact of infectious diseases or studies using health metrics other than YLL, YLD and/or DALY. For instance, we excluded studies that assessed potential years of life lost, and the probability of dying between an exact age-range as obtained from life tables. Moreover, we excluded studies that quantified disease burden attributable to risk factor exposure (e.g. outdoor air pollution, indoor smoke from solid fuel use, second-hand smoke, etc.). We also excluded letters to editor, editorials, correspondence or comments, as they lacked sufficient detail on characteristics and methodologies.

### Data screening and extraction

Two researchers (PC and VG) listed all the records obtained from the grey literature searches, reference checks and the burden-eu members, on an Excel spreadsheet. PC manually imported all these records to the EndNote X9 library provided by the Erasmus MC. After removing duplicates, PC and VG independently reviewed the resulting article titles (step 1) and abstracts (step 2) against the inclusion and exclusion criteria mentioned above. After this exclusion, PC and VG subsequently identified and screened potentially relevant burden of infectious disease studies upon full-text (step 3). When the two researchers disagreed on whether to include or exclude an article, they discussed their doubts with JH and LSJ.

Two researchers (PC and LSJ) critically appraised burden of infectious disease studies written in English using an adapted data extraction form developed for an earlier systematic review [24]. For studies written in languages that could not be read by any of the reviewers, PC organised online meetings with native speakers to perform the data extraction. We extracted information relating to the following items: study characteristics (i.e. author, title, aim, year of publication, infectious disease category studied, reference country/region included) as well as methodological approaches used to calculate YLL (i.e. choice of life table) and/or YLD (i.e. incidence- *vs.* prevalence-based approach and pathogen- *vs.* outcome-based approach, disability weights and social weighting functions). Definitions of the extracted items can be found in the Supplementary Material. PC and LSJ compared, assessed and discussed the final version of the completed data extraction form. Discussions with JH resolved any possible doubts.

### Synthesis of findings

We classified studies according to their study characteristics (e.g. year of publication, geographical coverage and infectious disease covered). Studies performed within a single-country of the EU/EEA/EFTA are referred to as ‘single-country’, whereas those that covered more than one country are referred to as ‘multi-country’. Studies estimating the burden of, for example, food-borne pathogens (e.g. *Salmonella* spp. and/or shiga-toxin producing *Escherichia coli* O157) were classified in the ‘food- and water-borne diseases’ group. Studies estimating the burden of multiple infectious diseases (e.g. hepatitis C, psittacosis), where none was preventable by a vaccine, were classified in the ‘other’ group. Further details about the data synthesis can be found in the Supplementary Material. We adopted the same approach for a total of eight infectious-specific groups, namely ‘COVID-19’, ‘food- and water-borne diseases’, ‘healthcare-associated infections’, ‘respiratory infections’, ‘sexually transmitted infections’, ‘vaccine-preventable diseases’, ‘zoonotic diseases’ and ‘other’. Definitions of these categories can also be found in the Supplementary Material.

## Results

### Literature search

**Fig. 1.**
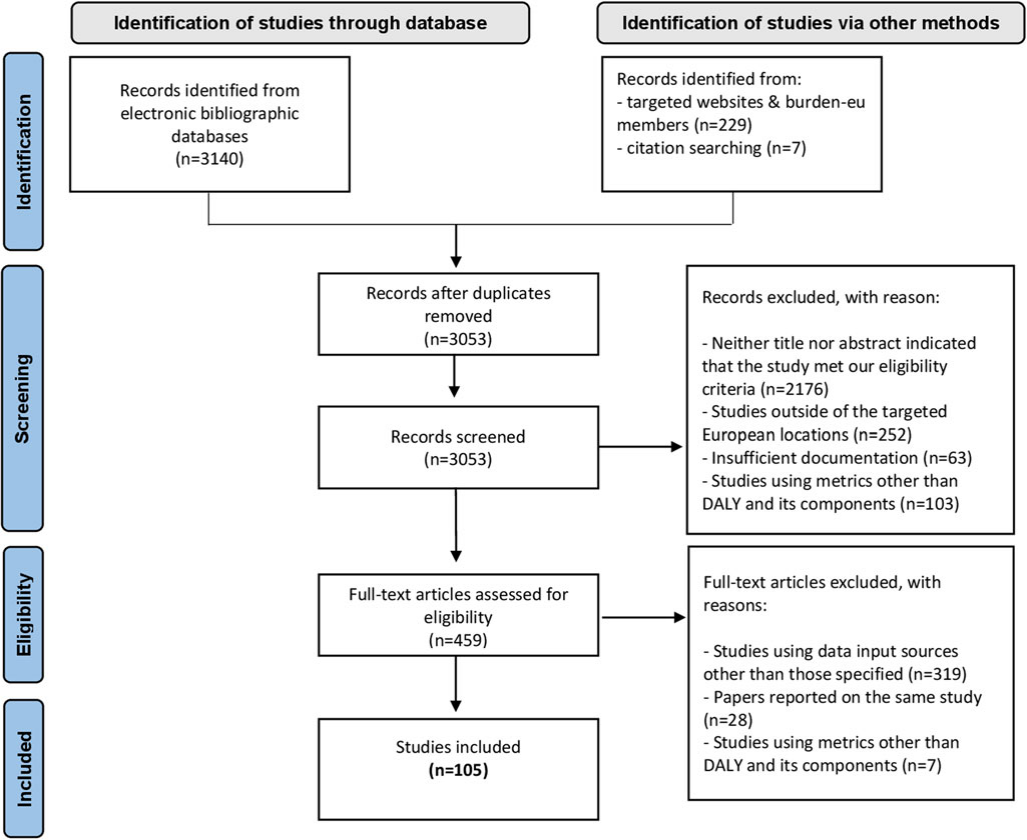
Flowchart of the literature search and study selection.

We identified 3376 records through database, grey literature and citation searching. After removing duplicates (*n* = 323), we screened titles and abstracts from 3053 records. We assessed 459 full-text articles for eligibility having excluded 354 studies because they did not meet the inclusion criteria: 319 were excluded because other data input sources than those we specified were used; 28 were excluded because they reported results from the same study; and seven because other health metrics than YLL, YLD and/or DALY were used to express the burden of disease. Finally, we extracted information from 105 burden of infectious disease studies (Fig. 1).

### Study characteristics

**Fig. 2.**
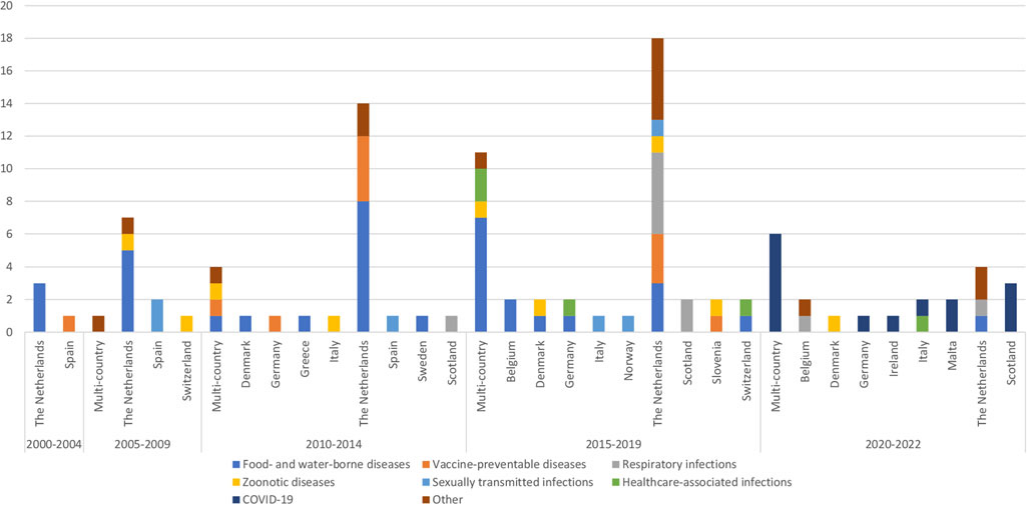
Number of single-country and multi-country independent burden of infectious disease studies per year of publication, geographic coverage and infectious cause category studied.

Of the 105 burden of infectious disease studies included for review, 22 and 83 were performed at a multi- and single-country level, respectively (Fig. 2).

The number of single-country studies increased by 300% in the overall study period, from four (the Netherlands, Spain) in 2000–2004 to 16 (Belgium, Denmark, Germany, Ireland, Italy, Malta, the Netherlands, Scotland) in 2020–2022 (Fig. 2). Over the 2000–2022 period, the largest number of studies was in the Netherlands (*n* = 46), with food- and water-borne diseases being the most studied (*n* = 15). Two EU/EEA/EFTA countries (Malta, Ireland) published their first independent infectious-specific calculations in the 2020–2022 period; these studies estimated the burden of COVID-19. The category of infectious disease that was studied also varied by country. Some countries (Belgium, Denmark, Italy, the Netherlands, Slovenia, Switzerland) estimated the burden of zoonotic diseases, whereas three countries (Germany, Italy, Switzerland) estimated the burden of healthcare-associated infections at a single-country level (Fig. 2).

The highest number of multi-country studies (*n* = 11) was seen in the 2015–2019 period; seven out of these 11 studies estimated the burden of food- and water-borne diseases. During the 2020–2022 period, the number of multi-country studies (*n* = 6) that estimated the burden of COVID-19 was slightly lower compared to those performed at a single-country level (*n* = 8).

### Methodological approaches for estimating YLL

#### Choice of life table

**Fig. 3.**
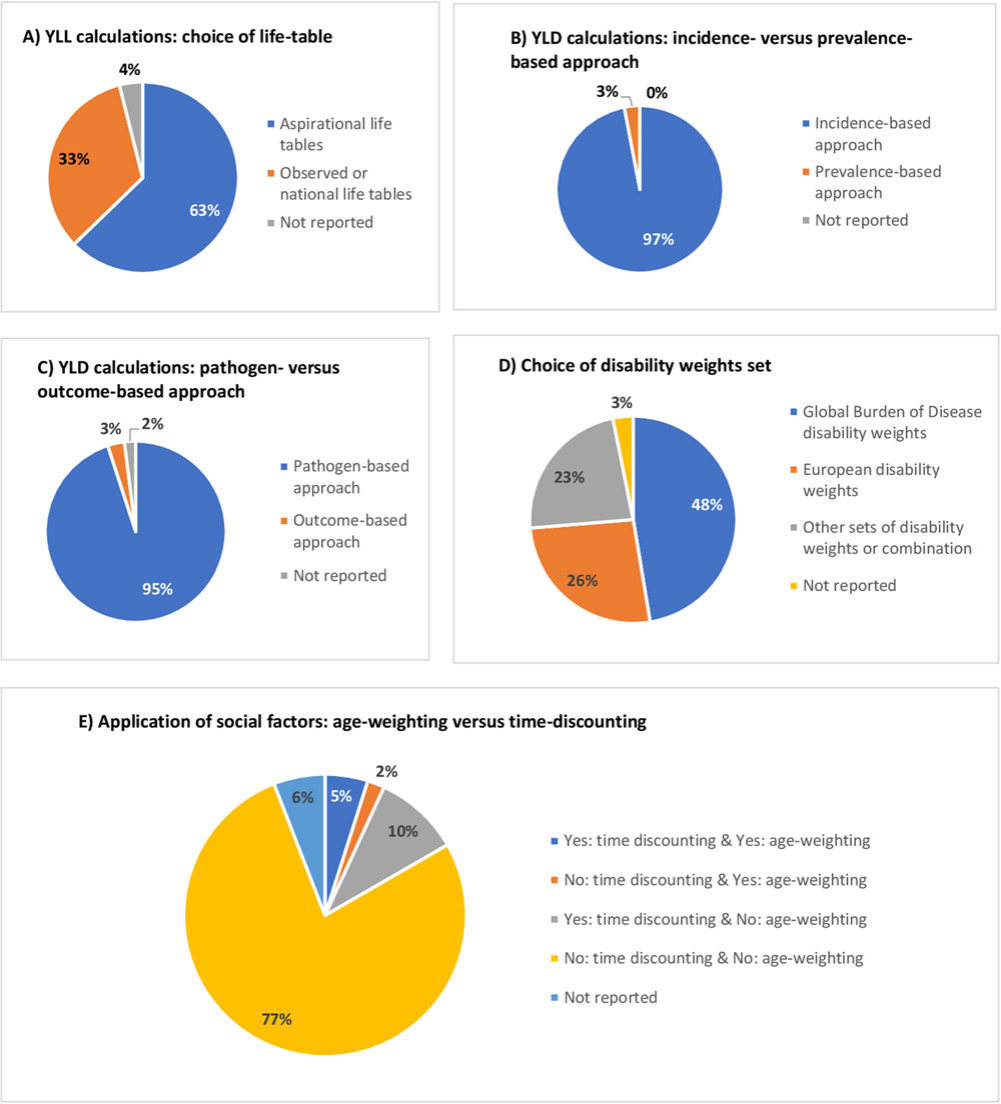
Methodological approaches used to estimate YLL and YLD in independent burden of infectious disease studies, 2000–2022*. *Proportions for burden of infectious disease studies that included years of life lost calculations were reported for 102 out of 105 studies, while for those included years lived with disability were reported for 95 out of 105 studies.

In total, 102 out of the 105 included studies estimated YLL. More than half of these studies (63%) used aspirational life tables (i.e. GBD or WHO) to estimate YLL, whereas 33% used national or regional life tables. Four studies did not report the life table that was used to estimate YLL (Fig. 3a).

### Methodological approaches for estimating YLD

#### Incidence- *vs.* prevalence-based approach

In total, 95 out of the 105 included studies estimated YLD. Ninety-two studies (97%) estimated YLD based on the incidence approach (Fig. 3b). A large proportion of these incidence-based studies estimated YLD for food- and water-borne diseases (39%) (Supplementary Material). Three studies estimated YLD using the prevalence-based approach (Fig. 3b). These studies assessed the burden of sexually transmitted infections, namely hepatitis B and/or hepatitis C viruses (*n* = 2) and HIV/AIDS (*n* = 1) (Supplementary Material).

#### Pathogen- *vs.* outcome-based approach

Among the studies that performed incidence-based YLD calculations, the vast majority (97%) applied a pathogen-based approach, while two out of these studies did not report the approach used (Supplementary Material and Fig. 3c). All studies that performed prevalence-based YLD calculations applied an outcome-based approach (3%) (Fig. 3c).

#### Choice of disability weights

Most studies (48%) used the GBD disability weights. Also, 26% of the included studies used the European set of disability weights as recommended and applied by the BCoDE study; these studies were published after 2015 (Supplementary Material). Some studies used other disability weight sets such as the Dutch disability weights (20%), or a combination of existing disability weight sets (3%). The percentage of studies that applied Dutch disability weights decreased after the elicitation of the European disability weights in 2015, from 18% in the 2000–2014 period to 4% in the 2015–2022 period (Supplementary Material). Three studies (3%) did not report the disability weights that were used (Fig. 3d).

#### Social weighting functions: use of age-weighting and time-discounting

Most of the identified studies (77%) did not apply age-weighting or time-discounting in their DALY estimations, while 17% applied age-weighting or time-discounting. Among studies that did, 10 (56%) performed scenario analyses by changing the rates of time-discounting, either by setting it to 0% (i.e. no time discounting), to 1.5% or to 3%. Most of these studies (*n* = 7) were conducted in the Netherlands and were in line with the Dutch guidelines for health economic evaluations [30, 31]. Five studies used both age-weighting and time-discounting; out of these studies, four were published before 2011 and one in 2021 (Supplementary Material). Two studies applied age-weighting and no time-discounting functions, one study published in 2010 and the other in 2020 (Supplementary Material). Six studies did not report whether social weighting values had been applied in their estimations (Fig. 3e).

## Discussion

### Summary of findings and interpretation of results

This systematic literature review aimed to provide insights into the characteristics and DALY-specific methodological design choices that have been made in independent burden of infectious disease studies undertaken in EU/EEA/EFTA countries and the United Kingdom. In total, 105 studies met our inclusion criteria and were included for review. We observed that 15 out of the 32 targeted countries published disease burden estimates for infectious diseases at a single-country level. Over the 2000–2022 period, most studies were conducted in the Netherlands. Dutch public health experts have estimated national and sub-national disease burden estimates for a variety of infectious diseases, thereby introducing the DALY concept as a standard metric to their epidemiological research [36]. In particular, the Center for Infectious Disease Control at the National Institute for Public Health and the Environment in the Netherlands (RIVM) has performed DALY calculations of infectious diseases on a routine basis by adjusting the parameter values for disease models in the BCoDE toolkit (i.e. a stand-alone software tool which allows estimation of DALYs for a number of infectious diseases) to better reflect the Dutch epidemiological situation [12, 37]. It should be noted that a potential limitation of using the exact same methodology to assess DALYs is that there is a risk of systematic under-estimation or over-estimation of the burden of a certain infectious disease. Each methodological choice has an impact on the resulting DALY estimation and by choosing a uniform methodology (e.g. disability weights, life expectancy) or epidemiological model (e.g. disease model, severity level distribution), these choices may not reflect the disease agent or study population, and as a result the DALY estimates will not reflect the true burden of disease.

We observed that some countries belonging to Central and Eastern Europe performed a small number of or no independent burden of infectious disease studies. This may in part reflect the limited resources for infectious disease research in many Eastern European countries. Another possible explanation may be that the quality of surveillance systems across European countries varies greatly, especially in terms of data availability, accessibility (e.g. patient characteristics, causative agent, number of cases and their severity, etc.) and timeliness of reporting [38, 39]. This can lead to challenges with accurate and timely reporting of the frequency, incidence and/or mortality of infectious diseases, particularly if these diseases are not notifiable in the country.

During the 2020–2022 period, the number of COVID-19 disease burden studies was 1.6 times higher compared to the number of studies estimating the burden of other infectious-related diseases. This focus was due to the alarming surge in COVID-19 cases. However, the focus on COVID-19 may have led to a decrease in the number of conducted, and thus documented, disease burden studies for other infectious diseases, such as sexually transmitted infections. The low number of burden of studies estimating the burden of sexually transmitted infections may be explained by heterogeneity in the coverage, completeness and repetitiveness of such data, previously reported across national reporting systems of most of the European countries [40, 41].

Another noteworthy observation was that almost all burden of COVID-19 studies followed consensus methodologies to estimate the impact of COVID-19 in EU/EEA/EFTA countries and the United Kingdom. The harmonisation of methods is attributable to the work of the COVID-19 Task Force of the burden-eu network, which aimed to support network members to estimate COVID-19 DALYs (42). The Task Force did this by, for instance, developing an open access protocol (available at: https://www.burden-eu.net/) providing guidance for researchers planning to estimate COVID-19 DALYs, which likely facilitated the number of burden of COVID-19 disease studies undertaken across Europe [42–44]. In addition, it led to the harmonisation of design choices and align strategies that need to be made when estimating the burden of COVID-19. Such practical and educational tools are crucial for burden of disease research, because they considerably enhance the comparability of disease burden estimates. Briefly, all burden of COVID-19 disease studies estimated YLDs based on the incidence- and pathogen-based approach, using health states descriptions and disability weights from the GBD and/or European disability weights measurement studies, and YLLs based on aspirational life table standards. None of these studies applied age-weighting and time-discounting to estimate the impact of COVID-19 [42]. Such consistencies in design choices produce comparable disease burden estimates and, in turn, allow for a quantification of the incurred disease burden, despite the preventative public health measures that were in place, adherence to these measures and available treatments. We therefore recommend the further development and use of protocols for performing burden of disease studies beyond COVID-19.

Furthermore, we observed that almost all burden of infectious disease studies estimating incidence-based YLDs have predominantly applied a pathogen-based approach and used aspirational life tables. With the incidence- and pathogen-based approach, the incidence of infectious diseases from a specific pathogen in a certain year is linked to all related potential health outcomes via a disease progression model (i.e. a schematic qualitative overview of the progression of an infection and its conditional frequency of occurrence in time). This allows for the estimation of the burden of those diseases considering the impact of different possible health outcomes, from acute and short-duration to long-term and/or late-onset sequelae. Therefore, to gain insight into the number of DALYs that can be averted by preventing a certain infectious disease, the pathogen- and incidence-based approach might be preferable to the prevalence-based approach when assessing YLD for infectious diseases. Based on this, we recommend that for future burden of infectious disease calculations a pathogen- and incidence- based approach is used. However, this may not be the preferred approach for infectious diseases that can have a duration of several years or even decades (e.g. certain sexually transmitted infections) and as such can be considered a chronic disease (e.g. hepatitis B, HIV/AIDS) [45, 46].

Linked to this choice is the use of aspirational life tables, as the pathogen- and incidence-based DALY estimates reflect current and future health loss due to a certain pathogen. However, using current (national) life tables to assess future health loss might lead to an underestimation of pathogen- and incidence-based infectious disease DALYs. Hence, aspirational life tables should be considered as the gold standard for pathogen- and incidence-based DALYs [42]. Although aspirational life tables are based on aspirational mortality risks that may differ from those currently observed in various countries, they greatly facilitate comparisons with other diseases and injuries, and between different countries and across time periods. Aspirational life tables also have important ethical advantages (on grounds of equity), as they assume the same remaining life expectancy values for both males and females [47, 48]. We therefore recommend that burden of infectious disease studies that employ the pathogen- and incidence-based approach use aspirational life tables to assess DALYs.

We observed that, over the years, social weighting values have explicitly been omitted from most burden of infectious disease studies. However, several studies from the Netherlands present both undiscounted and discounted infectious disease DALYs. The time-discounting concept discounts the years of (healthy) life that would have been lived in the future at a rate of, for example, 3%. However, larger or smaller differences might be seen with other burden of disease estimates [26, 49].

The shift from the Dutch disability weights [50] to the European disability weights [25] for infectious YLD calculations, especially in disease burden studies published after 2015, is another noteworthy finding of this review. This shift can primarily be explained by the fact that the Dutch disability weights [50] were derived in the 1990s and since then, the methods for deriving disability weights have evolved [22]. Differences in methodologies to derive disability weights have an impact on the actual value of disability weights, thereby inhibiting comparability with other burden of disease studies, as well as the validity and reproducibility of disability weights. Over the years, new sets of disability weights have been derived based on newer techniques, including the set of European disability weights [22, 25]. However, there were some variations in disability weights between and within European countries [51]. For future burden of infectious disease studies undertaken in Europe, we recommend that the European set of disability weights are used, since they are derived based on the most recent elicitation techniques and cover a wide range of infectious disease-related health states. Methodological design choices of burden of infectious disease studies are more consistent compared to those that have been used in non-communicable and injury burden of disease studies [29, 30]. An explanation for this finding may be that most of the studies that were included in our review were from the same country and mostly the same research teams. Furthermore, the BCoDE and WHO/FERG studies and their deliverables, including reports that explain the DALY methodological choices [18–20] and a calculation and reporting toolkit [12], may have facilitated harmonisation of infectious disease burden methods. The development of a guide to estimate COVID-19 DALYs was also crucial and in turn advanced harmonisation and quality of reporting of the burden of COVID-19 studies [44].

### Strengths and limitations

Although we have reviewed a variety of electronic databases, platforms and search engines; grey literature searches may have been limited. Nevertheless, independent burden of infectious disease studies in EU/EEA/EFTA countries and the United Kingdom have been identified and categorised by study characteristics resulting in an overview of DALY-specific methodological design choices that were used in burden of infectious disease studies over the period from 2000 to 2022. Moreover, in contrast to what is systematically performed in literature review, we did not perform a risk of bias assessment of the included burden of infectious disease studies, since the existing assessment tools were not suitable for evaluating the quality of burden of disease studies.

## Conclusions

The number of independent burden of infectious disease studies across Europe increased over time. The most studied infectious diseases were food- and water-borne-related diseases, with the Netherlands publishing the highest number of these studies. In Eastern Europe a very low number of burden of infectious disease studies have been performed, underlining that there is a merit in improving surveillance, data collection and capacity building. Moreover, disease models should be improved for infectious diseases with higher burden, as well as those infectious disease that are less well studied. The high consistency in methodological design choices highlights the importance of burden of disease tools and guidelines. The European Burden of Disease Network aims to develop reporting guidelines for conducting burden of disease studies.

## Data Availability

Not applicable.

## Supplementary material

For supplementary material accompanying this paper visit http://doi.org/10.1017/S0950268823000031. click here to view supplementary material
