# Supplementary material for: Burden of infectious disease studies in Europe and the United Kingdom: a review of methodological design choices
Source: Epidemiol Infect. 2023 Jan 9;151:e19. doi: 10.1017/S0950268823000031 (PMC9990389; doi:10.1017/S0950268823000031)
Supplement: Supplementary file 1 [file S0950268823000031sup001.docx]

**Supplementary Material**

**Burden of infectious disease studies in Europe and the United Kingdom: a review of methodological design choices**

**Chapter 1**

- 1. Selection of countries …………………………………………………..……………………………………………….………….. 2
  2. Classification of independent burden of infectious disease studies .……….…………………………………. 2
  3. Search strategy ………………………………………………………………………………………………………………………….. 5
  4. Grey literature search and websites of targeted national public health agencies ……….……………… 7
  5. Definitions of the used data extraction items ……………………………………….………………………………….… 8

**Chapter 2**

Preferred Reporting Items for Systematic Reviews and Meta-analyses (PRISMA) Statement …………. 12

**Chapter 3**

Reference list of the included studies ……..………………………………………………………………………………………. 15

**Chapter 4**

Data extraction list of the included studies ……..…………………………………..…………………………………………. 21

**Chapter 5**

List of authors and affiliations ……..…………………………………………….………..…………………………………………. 25

**Chapter 1**

- 1. **Selection of countries**

In this systematic literature review, we included independent burden of infectious disease studies undertaken across the European Union (EU) member states and European Economic Area/European Free Trade Association (EEA/EFTA) countries and the United Kingdom.

Full list of the included countries: *Austria, Belgium, Bulgaria, Croatia, Republic of Cyprus, Czech Republic, Denmark, Estonia, Finland, France, Germany, Greece, Hungary, Iceland, Ireland, Italy, Latvia, Liechtenstein, Lithuania, Luxembourg, Malta, the Netherlands, Norway, Poland, Portugal, Romania, Slovakia, Slovenia, Spain, Sweden, Switzerland, and the United Kingdom.*

- 1. **Classification of independent burden of infectious disease studies**

We classified studies according to their study characteristics (e.g., year of publication, geographical coverage, and infectious disease covered). Studies performed within a single-country of the EU/EEA/EFTA are referred to as ‘single-country’, whereas those that covered more than one country are referred to as ‘multi-country’. Studies estimating the burden of, for example, food-borne pathogens (e.g., Salmonella spp. and/or shiga-toxin producing Escherichia coli O157) were classified in the ‘food- and water-borne diseases’ group. Studies estimating the burden of multiple infectious diseases (e.g., measles, hepatitis C, legionellosis) with at least one of these diseases can be prevented by a vaccine (e.g., measles) were classified in the ‘other’ group. Studies estimating the burden of multiple infectious diseases (e.g., hepatitis C, psittacosis), where none was preventable by a vaccine, were also classified in the ‘other’ group.

We adopted the same approach for a total of eight infectious-specific groups, namely ‘COVID-19’; ‘food- and water-borne diseases’; ‘healthcare-associated infections; ‘other’; ‘respiratory infections’; ‘sexually transmitted infections’; ‘vaccine-preventable diseases’; and ‘zoonotic diseases’.

We followed case definitions and examples as proposed in Burden of Communicable Diseases in Europe (BCoDE) project, the European Centre for Disease Prevention and Control (ECDC) website (<https://www.ecdc.europa.eu/en>), as well as World Health Organization (<https://www.who.int/>).

- **COVID-19:** COVID-19, the illness caused by SARS-CoV-2, emerged in late 2019 and spread very quickly across the globe. Within the first two years of the COVID-19 pandemic, more than 450 million cases were reported worldwide, more than 100 million in the EU/EEA alone. SARS-CoV-2 is mainly spread via respiratory droplets, including aerosols, from an infected person who sneezes, coughs, speaks, sings or breathes in close proximity to other people. Droplets can be inhaled or deposited in the nose and mouth or on the eyes.
- **Food- and water-borne diseases:** Food- and water-borne illness, often referred to as food poisoning, can be caused by eating or drinking food or beverages contaminated by bacteria, parasites or viruses. Most human foodborne diseases are caused by *Campylobacter*, *Salmonella*, *Yersinia*, *E. coli* and *Listeria* bacteria.
- **Healthcare-associated infections:** Infections acquired by patients during their stay in a hospital or another healthcare setting (e.g., surgical site infections, urinary tract infections, bloodstream infections and gastro-intestinal infections etc)
- **Vaccine-preventable diseases:** Diseases that can be prevented by vaccination (e.g., cholera, diphtheria, influenza A, Hepatitis B, human papillomavirus, haemophilus influenzae disease, invasive pneumococcal disease, Japanese encephalitis, measles, etc)
- **Sexually transmitted infections**: Infections that may transmit through vaginal, oral and anal sexual intercourse.
- **Zoonotic diseases:** Infectious disease that has jumped from a non-human animal to humans. Zoonotic pathogens may be bacterial, viral or parasitic, or may involve unconventional agents and can spread to humans through direct contact or through food, water or the environment
- **Other:** Other infectious-specific causes of disease that did not belong to the aforementioned categories specified by the authors. Also, studies estimating the burden of multiple infectious diseases where at least one of these diseases can be prevented by a vaccine (e.g., measles) were classified in the ‘other’ group.

| **Infectious-specific groups** | **Definition & Examples** |
| --- | --- |
| COVID-19 | Studies estimating the direct and/or indirect impact of COVID-19. |
| Food- and water-borne diseases | Studies estimating the burden of food- and/or water-borne diseases. However, if at least one vaccine-preventable foodborne disease was involved in a foodborne-specific study, it was then classified in this category and not in the vaccine-preventable one. |
| Healthcare-associated infections | Studies estimating the burden of healthcare-associated infections as proposed in the BCoDE project and ECDC. |
| Vaccine-preventable diseases | Studies estimating the burden of vaccine-preventable infections as proposed in the BCoDE project and ECDC. |
| Sexually transmitted infections | Studies estimating the burden of sexually transmitted infections as proposed in the BCoDE project and ECDC. However, if at least one vaccine-preventable sexually transmitted infection was involved in a sexually infections-specific study, it was then classified in this category and not in the vaccine-preventable one. |
| Respiratory infections | Studies estimating the burden of respiratory infections as proposed in the BCoDE project and ECDC. However, if at least one vaccine-preventable respiratory infection was involved in a respiratory infection-specific study, it was then classified in this category and not in the vaccine-preventable one. |
| Zoonotic diseases | Studies estimating the burden of zoonotic diseases as proposed in the BCoDE project and ECDC |
| Other | Studies estimating the burden of multiple infectious diseases (e.g., measles, hepatitis C, legionellosis) with at least one of these diseases can be prevented by a vaccine (e.g., measles) were classified in the ‘other’ group. Studies estimating the burden of multiple infectious diseases (e.g., hepatitis C, psittacosis), where none was preventable by a vaccine, were also classified in the ‘other’ group. |

- 1. **Search Strategy**

**Embase**

('disability-adjusted life year'/de OR (DALY OR DALYs OR ((disabil*) NEAR/4 (adjust*) NEAR/4 (life*) NEAR/4 (year*)) OR YLL OR YLLs OR ((year*) NEXT/2 (life*) NEXT/1 (lost*)) OR YLD OR YLDs OR ((year*) NEAR/3 (lived) NEAR/3 (disabil*))):ab,ti,kw) AND ('Europe'/exp OR 'Yugoslavia'/de OR 'Israel'/de OR 'European Union'/de OR 'European'/de OR 'EU citizen'/de OR (europ* OR austria* OR belgium OR belgian* OR Denmark OR danish OR france OR french* OR german* OR ireland OR irish* OR italy OR italian* OR luxemb* OR netherlands OR dutch OR norway OR sweden OR switzerland OR swiss OR united-kingdom OR albania OR armenia OR bosnia* OR herzegovin* OR bulgar* OR croatia* OR cyprus OR czechoslovakia* OR estonia* OR finland OR georgia OR greece OR hungar* OR iceland* OR israel* OR kosov* OR latvia* OR lithuan* OR macedoni* OR malta OR montenegr* OR poland OR polish OR portug* OR romani* OR rumani* OR serbi* OR slovak* OR sloven* OR spain* OR spanish OR turkey* OR mediterran* OR czech* OR england* OR UK OR scotland OR wales OR britain* OR holland* OR scandinav* OR nordic-countr* OR yugoslov* OR baltic* OR flander* OR wallon* OR benelux* OR greek* OR andorra* OR azerbaijan* OR belarus* OR byelarus* OR byelorus* OR white-russia* OR monaco* OR moldova* OR moldovia* OR russian-federat* OR san-marin* OR ukrain*):ab,ti,kw)

**Medline**

((DALY OR DALYs OR ((disabil*) ADJ4 (adjust*) ADJ4 (life*) ADJ4 (year*)) OR YLL OR YLLs OR ((year*) ADJ2 (life*) ADJ (lost*)) OR YLD OR YLDs OR ((year*) ADJ3 (lived) ADJ3 (disabil*))).ab,ti,kf.) AND (exp Europe/ OR Yugoslavia/ OR Israel/ OR European Union/ OR (europ* OR austria* OR belgium OR belgian* OR Denmark OR danish OR france OR french* OR german* OR ireland OR irish* OR italy OR italian* OR luxemb* OR netherlands OR dutch OR norway OR sweden OR switzerland OR swiss OR united-kingdom OR albania OR armenia OR bosnia* OR herzegovin* OR bulgar* OR croatia* OR cyprus OR czechoslovakia* OR estonia* OR finland OR georgia OR greece OR hungar* OR iceland* OR israel* OR kosov* OR latvia* OR lithuan* OR macedoni* OR malta OR montenegr* OR poland OR polish OR portug* OR romani* OR rumani* OR serbi* OR slovak* OR sloven* OR spain* OR spanish OR turkey* OR mediterran* OR czech* OR england* OR UK OR scotland OR wales OR britain* OR holland* OR scandinav* OR nordic-countr* OR yugoslov* OR baltic* OR flander* OR wallon* OR benelux* OR greek* OR andorra* OR azerbaijan* OR belarus* OR byelarus* OR byelorus* OR white-russia* OR monaco* OR moldova* OR moldovia* OR russian-federat* OR san-marin* OR ukrain*).ab,ti,kf.)

**Cochrane**

((DALY OR DALYs OR ((disabil*) NEAR/4 (adjust*) NEAR/4 (life*) NEAR/4 (year*)) OR YLL OR YLLs OR ((year*) NEXT/2 (life*) NEXT/1 (lost*)) OR YLD OR YLDs OR ((year*) NEAR/3 (lived) NEAR/3 (disabil*))):ab,ti) AND ((europ* OR austria* OR belgium OR belgian* OR Denmark OR danish OR france OR french* OR german* OR ireland OR irish* OR italy OR italian* OR luxemb* OR netherlands OR dutch OR norway OR sweden OR switzerland OR swiss OR united-kingdom OR albania OR armenia OR bosnia* OR herzegovin* OR bulgar* OR croatia* OR cyprus OR czechoslovakia* OR estonia* OR finland OR georgia OR greece OR hungar* OR iceland* OR israel* OR kosov* OR latvia* OR lithuan* OR macedoni* OR malta OR montenegr* OR poland OR polish OR portug* OR romani* OR rumani* OR serbi* OR slovak* OR sloven* OR spain* OR spanish OR turkey* OR mediterran* OR czech* OR england* OR UK OR scotland OR wales OR britain* OR holland* OR scandinav* OR nordic-countr* OR yugoslov* OR baltic* OR flander* OR wallon* OR benelux* OR greek* OR andorra* OR azerbaijan* OR belarus* OR byelarus* OR byelorus* OR white-russia* OR monaco* OR moldova* OR moldovia* OR russian-federat* OR san-marin* OR ukrain*):ab,ti)

**Web of Science**

TS=(((DALY OR DALYs OR ((disabil*) NEAR/4 (adjust*) NEAR/4 (life*) NEAR/4 (year*)) OR YLL OR YLLs OR ((year*) NEAR/2 (life*) NEAR/1 (lost*)) OR YLD OR YLDs OR ((year*) NEAR/2 (lived) NEAR/2 (disabil*)))) AND ((europ* OR austria* OR belgium OR belgian* OR Denmark OR danish OR france OR french* OR german* OR ireland OR irish* OR italy OR italian* OR luxemb* OR netherlands OR dutch OR norway OR sweden OR switzerland OR swiss OR united-kingdom OR albania OR armenia OR bosnia* OR herzegovin* OR bulgar* OR croatia* OR cyprus OR czechoslovakia* OR estonia* OR finland OR georgia OR greece OR hungar* OR iceland* OR israel* OR kosov* OR latvia* OR lithuan* OR macedoni* OR malta OR montenegr* OR poland OR polish OR portug* OR romani* OR rumani* OR serbi* OR slovak* OR sloven* OR spain* OR spanish OR turkey* OR mediterran* OR czech* OR england* OR UK OR scotland OR wales OR britain* OR holland* OR scandinav* OR nordic-countr* OR yugoslov* OR baltic* OR flander* OR wallon* OR benelux* OR greek* OR andorra* OR azerbaijan* OR belarus* OR byelarus* OR byelorus* OR white-russia* OR monaco* OR moldova* OR moldovia* OR russian-federat* OR san-marin* OR ukrain*)))

**Google Scholar**

"disability adjusted life years" europe|france|germany|italy|netherlands|norway|sweden|switzerland|"united kingdom"|finland|greece|hungaria|israel|poland|portugal|romania|spain|turkey|england|britain|Scandinavia

- 1. **Grey literature search and websites of targeted national public health agencies**

*Grey literature search engines*

| - OpenGrey: [www.opengrey.eu](http://www.opengrey.eu) | - **CABDirect:** [www.cabdirect.org](http://www.cabdirect.org) |
| --- | --- |
| - OAIster: <http://oaister.worldcat.org> | - **World Health Organization:** [www.who.int](http://www.who.int) |

*Websites of targeted national public health agencies*

| - Austria: [goeg.at/](https://goeg.at/) |  |
| --- | --- |
| - Belgium: <https://www.sciensano.be/en>; https://www.fasfc.be/ |  |
| - Bulgaria: [ncpha.government.bg](http://ncpha.government.bg) |  |
| - Croatia: <https://www.hzjz.hr/en/directorate/croatian-institute-of-public-health/> |  |
| - Republic of Cyprus: [www.moh.gov.cy/](http://www.moh.gov.cy/) |  |
| - Czech Republic: [www.szu.cz](http://www.szu.cz) |  |
| - Denmark: [www.si-folkesundhed.dk](http://www.si-folkesundhed.dk) |  |
| - Estonia: [www.tai.ee](http://www.tai.ee) |  |
| - Finland: [www.thl.fi/en/](http://www.thl.fi/en/) |  |
| - France: [www.santepubliquefrance.fr](http://www.santepubliquefrance.fr) |  |
| - Germany: [www.rki.de](http://www.rki.de/EN) |  |
| - Greece: [www.statistics.gr](http://www.statistics.gr); [www.eody.gov.gr](http://www.eody.gov.gr) |  |
| - Hungary: [www.nnk.gov.hu/](http://www.nnk.gov.hu/) |  |
| - Iceland: [www.landlaeknir.is](http://www.landlaeknir.is/) |  |
| - Ireland: [www.publichealth.ie](http://www.publichealth.ie/) |  |
| - Italy: [www.iss.it/](http://www.iss.it/)  - **Latvia:** [www.rsu.lv/en/institute-public-health](https://www.rsu.lv/en/institute-public-health)  - **Lithuania:** [sam.lrv.lt/en/](https://sam.lrv.lt/en/)  - **Luxembourg:** [www.lih.lu/](https://www.lih.lu/) |  |
| - **Malta:** [www.deputyprimeminister.gov.mt/](http://www.deputyprimeminister.gov.mt/) |  |
| - **Norway:** [www.fhi.no](http://www.fhi.no/) |  |
| - **Poland:** [www.pzh.gov.pl](http://www.pzh.gov.pl/) |  |
| - **Portugal:** [www.dgs.pt](http://www.dgs.pt); [www.sns.gov.pt](http://www.sns.gov.pt) |  |
| - **Romania:** [www.insp.gov.ro/](https://www.insp.gov.ro/) |  |
| - **Slovakia:** [www.uvzsr.sk/en/](http://www.uvzsr.sk/en/) |  |
| - **Slovenia:** [www.nijz.si](http://www.nijz.si/) |  |
| - **Spain:** [www.isciii.es](http://www.isciii.es) |  |
| - **Sweden:** [www.folkhalsomyndigheten.se](http://www.folkhalsomyndigheten.se/) |  |
| - **Switzerland:** [www.bag.admin.ch/bag/de/home.html](http://www.bag.admin.ch/bag/de/home.html) |  |
| - **United Kingdom (Scotland):** [www.gov.uk/government/organisations/public-health-england](https://www.gov.uk/government/organisations/public-health-england); [www.scotpho.org.uk/](http://www.scotpho.org.uk/) |  |

- 1. **Definitions of the data extraction items used**

| General Information | PMID (or doi) | A unique identifier number which assigned to a specific reference/article in the PubMed website. |
| --- | --- | --- |
|  | Journal | The name of the journal that published the selected article. |
|  | Title | The full title of the selected article. |
|  | Author(s) | List of author(s) with the use of Vancouver style |
|  | Year | The year that the selected burden of disease (BoD) study was published. |
|  | Language | The written language of the BoD study |
| Study characteristics | Cause of disease | A single disease related to infectious or non-communicable disease or injury or an aggregation of diseases and injuries.  i.e., Non-communicable diseases (NCDs) [Yes/No]; Infectious diseases [Yes/No]; Injuries [Yes/No]  Define as **infectious-specific** **BoD assessment** |
|  | Type of study | **Independent study** (i.e., single-country or multi-country studies that performed own calculations and analyses of years of life lost (YLL), years –lived with disability (YLD) and/or disability-adjusted life years (DALY) using primary data sources).  Define as **independent burden of infectious disease study** |
|  | Reference population | Population whose health causes during some period of time is the source of the study data. Define as **single-country** or **multi-country** |
|  | Reference year | The year for which an estimate of incidence/prevalence/BoD is reported. |
|  | Stratification | The specific cause(s), and/or disease(s), and/or risk factor(s) related to mortality or disability indicators stratified for each year, age, and sex. [Yes/No] |
| Data input sources  Data input sources  *(continued)* | Data source mortality/YLL | *Were data sources that were used to derive mortality/YLL data specified by the authors?* [Yes/No]  *Relevant approaches for mortality data:* National statistics, disease registries, registry of death, survey data, vital registration systems, verbal autopsies, death registration systems, published literature, etc. |
|  | Mortality/YLL:  data integration | *Were multiple data sources integrated to arrive at the mortality/YLL data?* [Yes/No] |
|  | Data source incidence/prevalence/YLD  Data source incidence/prevalence/YLD  *(continued)* | *Were data sources that were used to derive incidence/prevalence/YLD data specified by the authors?* [Yes/No]  *Relevant approach and data source for morbidity (burden of infectious disease studies)*:   - Laboratory-confirmed cases (e.g., surveillance data, etc.) - Cross-sectional data (e.g., cohort or cross-sectional studies, etc.) - Syndrome surveillance or survey data (e.g., population data, etc.) - Exposure data (e.g., population exposure data, etc.)   *Other relevant data sources for morbidity data*  Published literature, disease registries, routine administrative and survey datasets, surveillance systems, health facility data, etc |
|  | Incidence/prevalence/YLD: data integration | *Were multiple data sources integrated to arrive at the incidence/prevalence/YLD data?* [Yes/No] |
| Data adjustments | Mortality/YLL:  data adjustment | [Yes/No] |
|  | Incidence/prevalence/YLD: data adjustment | [Yes/No] |
|  | Internal consistency | *Were adjustments made to ensure that the sum of cause‐specific mortality or impairments equals all‐cause mortality or impairments?* |
|  | Use of DisMod | DisMod is s a software tool that may be used to check the consistency of estimates of incidence, prevalence, duration and case fatality for diseases.  *Did the authors mention the use of DisMoD?* [Yes/No] |
| DALY method  DALY method  *(continued)* | Perspective of YLD estimates | - *Prevalence-based* approach takes point prevalence measures of disability, adjusted for seasonal variation. - *Incidence-based* approach captures the BoD in new diagnostic cases during a reference time-period and links all possible sequelae in future through an outcome tree or disease progression model. |
|  | Data pathway used | *How incidences of the individual health states were obtained?*   - *Direct approach*: direct incidence estimates of the infectious-associated health outcome(s) - *Attribution approach*: starting from the overall incidence of health outcomes, the incidence of a specific health state is obtained using attributable proportion to infectious diseases - *Transition approach*: starting from the overall incidence of infectious diseases, the incidence of a specific health state is obtained using transition probabilities/distributions |
|  | Life expectancy for YLL | The life table that was used to assess YLL  *Relevant life-tables*: Aspirational standard life tables, i.e., WHO standard life table, GBD standard life table OR National life tables or national life expectancy |
|  | Disease model | A disease model is a causal chain of a disease that describes health states and their transition probabilities over time. *Did the authors report the disease model that they have used to assess BoD?* [Yes/No] |
|  | Hazard or outcome, or risk factor-based approach | Approaches that the incidences of health states in the disease model, were obtained:   - *Outcome-based approach:* represents different health states of diseases, irrespective of the possible (infectious or non-infectious) aetiologies - *Hazard-based approach*: represents different health states associated with hazards (i.e., biological or chemical agents or traumas) - *Risk factor-based approach:*  represent different health states associated with risk factors |
|  | DW: source | The source of the set(s) of disability weights (DWs) that were used to assess YLD.  *Relevant sources*: GBD DWs, Dutch DWs, Empirical DWs etc. |
|  | DW: elicitation method  (only if study developed own DWs) | Methods for eliciting health state valuations  *Relevant methods*: Visual Analogue Scale (VAS), Person Trade-Off (PTO), Time Trade-Off (TTO), etc. |
|  | DW: panel of judges  (only if study developed own DWs) | The panel of judges whose preferences were obtained to assess DWs  *Relevant panel composition*: medical experts, healthcare professionals, policymakers, patients or people with disabilities, patients’ families, etc. |
|  | DW: severity distribution | The proportion of cases with e.g., mild, moderate or severe health state^1^ of a specific outcome for which separate DWs are available.  *Was a severity distribution used/reported by the authors?* [Yes/No]; [Global/National]  ^1^: a health state reflects a combination of signs or symptoms that result in a certain amount of health loss |
|  | Comorbidity adjustment  (YLD calculation) | Adjustment of YLD data for comorbidity^2^ [Yes/No]  ^2^: multiple conditions co-existing in one individual |
|  | Methods co-morbidity adjustment  (YLD calculation) | - Approach(es) used to deal with the impact of comorbidity in a BoD study e.g., Standard simulation method, etc  - Approach(es) that can be used to adjust DW’ data for comorbidity  e.g., Additive approach, Multiplicative approach, Maximum limit approach etc |
|  | Social weighting:  age weighting | By incorporating age-weighting into DALY implies that the value of life depends on age; a lower weight of healthy life years lived is given at younger and at older ages – known as ‘non-uniform DALY’ [Yes/No] |
|  | Social weighting:  time discounting | Time-discounting discounts future years of healthy life lived using a rate of 3% or an alternative set of 0% [Yes/No] |
|  | Social weighting: discounting rate | ‘*uniform DALY’*; age-weighting and 3% time-discounting rate; ‘*non-uniform DALY’*; no age-weighting, no time-discounting rate; ‘*age-weighting DALY’*; age-weighting, no time-discounting rate; ‘*time-discounting DALY’*; no age-weighting, 3% time-discounting rate [%] |
| Uncertainty | Uncertainty analysis | An estimation of range or distribution of uncertainty in estimates based on an assessment of the uncertainty or confidence intervals for all data and parameter inputs [Yes/No] |
|  | Uncertainty analysis: method | *Relevant methods of uncertainty in DALY calculations:* Parameter uncertainty, Structural or model uncertainty, Methodological uncertainty |
|  | Sensitivity analysis | Analysis of how the impact of uncertainties of one or more input variables can lead to uncertainties in data inputs or assumptions [Yes/No] |
|  | Scenario analysis | The current or future disease burden is compared with the BoD if one element is changed (e.g., life expectancy, DW or severity distribution) [Yes/No] |
|  | Scenario analysis: element changed | Element that was changed for the scenario analysis (e.g., life expectancy, DW or severity distribution) |
| BoD: burden of disease; DALY: disability adjusted life years; DW: disability weight; GBD: Global Burden of Disease; PMID: PubMed Identifier; YLD: years lost due to disability; YLL: years of life lost due to premature mortality; WHO: World Health Organisation.  Please note that items in red-colored text were extracted and critically discussed in this systematic literature review. | | |

**Chapter 2**

| **Section and Topic** | **Item #** | **Checklist item** | **Location where item is reported** |
| --- | --- | --- | --- |
| **TITLE** | | |  |
| Title | 1 | Identify the report as a systematic review. | 1 |
| **ABSTRACT** | | |  |
| Abstract | 2 | See the PRISMA 2020 for Abstracts checklist. | 189/203 |
| **INTRODUCTION** | | |  |
| Rationale | 3 | Describe the rationale for the review in the context of existing knowledge. | 218/295 |
| Objectives | 4 | Provide an explicit statement of the objective(s) or question(s) the review addresses. | 278/295 |
| **METHODS** | | |  |
| Eligibility criteria | 5 | Specify the inclusion and exclusion criteria for the review and how studies were grouped for the syntheses. | 316/332 |
| Information sources | 6 | Specify all databases, registers, websites, organisations, reference lists and other sources searched or consulted to identify studies. Specify the date when each source was last searched or consulted. | 304/314 |
| Search strategy | 7 | Present the full search strategies for all databases, registers and websites, including any filters and limits used. | 304/314 |
| Selection process | 8 | Specify the methods used to decide whether a study met the inclusion criteria of the review, including how many reviewers screened each record and each report retrieved, whether they worked independently, and if applicable, details of automation tools used in the process. | 334/353 |
| Data collection process | 9 | Specify the methods used to collect data from reports, including how many reviewers collected data from each report, whether they worked independently, any processes for obtaining or confirming data from study investigators, and if applicable, details of automation tools used in the process. | 334/353 |
| Data items | 10a | List and define all outcomes for which data were sought. Specify whether all results that were compatible with each outcome domain in each study were sought (e.g. for all measures, time points, analyses), and if not, the methods used to decide which results to collect. | NA |
|  | 10b | List and define all other variables for which data were sought (e.g. participant and intervention characteristics, funding sources). Describe any assumptions made about any missing or unclear information. | NA |
| Study risk of bias assessment | 11 | Specify the methods used to assess risk of bias in the included studies, including details of the tool(s) used, how many reviewers assessed each study and whether they worked independently, and if applicable, details of automation tools used in the process. | NA |
| Effect measures | 12 | Specify for each outcome the effect measure(s) (e.g. risk ratio, mean difference) used in the synthesis or presentation of results. | NA |
| Synthesis methods | 13a | Describe the processes used to decide which studies were eligible for each synthesis (e.g. tabulating the study intervention characteristics and comparing against the planned groups for each synthesis (item #5)). | 355/367 |
|  | 13b | Describe any methods required to prepare the data for presentation or synthesis, such as handling of missing summary statistics, or data conversions. | 355/367 |
|  | 13c | Describe any methods used to tabulate or visually display results of individual studies and syntheses. | 355/367 |
|  | 13d | Describe any methods used to synthesize results and provide a rationale for the choice(s). If meta-analysis was performed, describe the model(s), method(s) to identify the presence and extent of statistical heterogeneity, and software package(s) used. | 355/367 |
|  | 13e | Describe any methods used to explore possible causes of heterogeneity among study results (e.g. subgroup analysis, meta-regression). | NA |
|  | 13f | Describe any sensitivity analyses conducted to assess robustness of the synthesized results. | NA |
| Reporting bias assessment | 14 | Describe any methods used to assess risk of bias due to missing results in a synthesis (arising from reporting biases). | NA |
| Certainty assessment | 15 | Describe any methods used to assess certainty (or confidence) in the body of evidence for an outcome. | NA |
| **RESULTS** | | |  |
| Study selection | 16a | Describe the results of the search and selection process, from the number of records identified in the search to the number of studies included in the review, ideally using a flow diagram. | 370/377 |
|  | 16b | Cite studies that might appear to meet the inclusion criteria, but which were excluded, and explain why they were excluded. | NA |
| Study characteristics | 17 | Cite each included study and present its characteristics. | NA |
| Risk of bias in studies | 18 | Present assessments of risk of bias for each included study. | NA |
| Results of individual studies | 19 | For all outcomes, present, for each study: (a) summary statistics for each group (where appropriate) and (b) an effect estimate and its precision (e.g. confidence/credible interval), ideally using structured tables or plots. | 380/398 |
| Results of syntheses | 20a | For each synthesis, briefly summarise the characteristics and risk of bias among contributing studies. | 380/398 |
|  | 20b | Present results of all statistical syntheses conducted. If meta-analysis was done, present for each the summary estimate and its precision (e.g. confidence/credible interval) and measures of statistical heterogeneity. If comparing groups, describe the direction of the effect. | NA |
|  | 20c | Present results of all investigations of possible causes of heterogeneity among study results. | 400/442 |
|  | 20d | Present results of all sensitivity analyses conducted to assess the robustness of the synthesized results. | 400/442 |
| Reporting biases | 21 | Present assessments of risk of bias due to missing results (arising from reporting biases) for each synthesis assessed. | 400/442 |
| Certainty of evidence | 22 | Present assessments of certainty (or confidence) in the body of evidence for each outcome assessed. | NA |
| **DISCUSSION** | | |  |
| Discussion | 23a | Provide a general interpretation of the results in the context of other evidence. | 445/543 |
|  | 23b | Discuss any limitations of the evidence included in the review. | 545/553 |
|  | 23c | Discuss any limitations of the review processes used. | 545/553 |
|  | 23d | Discuss implications of the results for practice, policy, and future research. | 445/543 |
| **OTHER INFORMATION** | | |  |
| Registration and protocol | 24a | Provide registration information for the review, including register name and registration number, or state that the review was not registered. | 302 |
|  | 24b | Indicate where the review protocol can be accessed, or state that a protocol was not prepared. | 302 |
|  | 24c | Describe and explain any amendments to information provided at registration or in the protocol. | 302 |
| Support | 25 | Describe sources of financial or non-financial support for the review, and the role of the funders or sponsors in the review. | 575 |
| Competing interests | 26 | Declare any competing interests of review authors. | 572 |
| Availability of data, code and other materials | 27 | Report which of the following are publicly available and where they can be found: template data collection forms; data extracted from included studies; data used for all analyses; analytic code; any other materials used in the review. | 569 |

**Chapter 3**

| **Author** | **Year of Publication** | **Title of the study** |
| --- | --- | --- |
| Becker et al | 2020 | Superficial mycoses in Belgium: Burden, costs and antifungal drugs consumption |
| Bermudez-Tamayo et al | 2008 | Factors associated with improvement in disability-adjusted life years in patients with HIV/AIDS |
| Bordino et al | 2021 | Burden of Healthcare-Associated Infections in Italy: Disability-Adjusted Life Years |
| Brooke et al | 2014 | Comparing the impact of two concurrent infectious disease outbreaks on The Netherlands population, 2009, using disability-adjusted life years |
| Cassini et al | 2016 | Impact of food and water-borne diseases on European population health |
| Cassini et al | 2016 | Burden of Six Healthcare-Associated Infections on European Population Health: Estimating Incidence-Based Disability- Adjusted Life Years through a Population Prevalence-Based Modelling Study |
| Cassini et al | 2018 | Impact of infectious diseases on population health using incidence-based disability-adjusted life years (DALYs): results from the Burden of Communicable Diseases in Europe study, European Union and European Economic Area countries, 2009 to 2013 |
| Cassini et al | 2019 | Attributable deaths and disability-adjusted life-years caused by infections with antibiotic-resistant bacteria in the EU and the European Economic Area in 2015: a population-level modelling analysis |
| Colzani et al | 2014 | Impact of measles national vaccination coverage on burden of measles across 29 Member States of the European Union and European Economic Area, 2006–2011 |
| Cortés et al | 2004 | Carga de enfermedad atribuible a las afecciones inmunoprevenibles en la población infantojuvenil española |
| Cuschieri et al | 2021 | Estimating the direct Covid-19 disability-adjusted life years impact on the Malta population for the first full year |
| Cuschieri & Grech | 2021 | Protecting our vulnerable in the midst of the COVID-19 pandemic: lessons learnt from Malta |
| Cuschieri et al | 2022 | Mortality comparisons of COVID-19 with all-cause and non-communicable diseases in Cyprus, Iceland and Malta: lessons learned and forward planning |
| Devleesschauwer et al | 2015 | The low global burden of trichinellosis: evidence and implications |
| Farangel et al | 2017 | Estimating the annual burden of tick-borne encephalitis to inform vaccination policy, Slovenia, 2009 to 2013 |
| Fastl et al | 2020 | The burden of legionnaires' disease in Belgium, 2013 to 2017 |
| Fan et al | 2021 | Estimating global burden of COVID-19 with disability-adjusted life years and value of statistical life metrics |
| Gasser et al | 2019 | Attributable deaths and disability-adjusted life-years caused by infections with antibiotic-resistant bacteria in Switzerland |
| Garcia-Fulgueiras et al | 2009 | Hepatitis C and hepatitis B-related mortality in Spain |
| Garcia-Fulgueiras et al | 2011 | Burden of disease related to hepatitis C and hepatitis B in Spain: a methodological challenge of an unfolding health problem |
| Gaunt et al | 2011 | Disease burden of the most commonly detected respiratory viruses in hospitalized patients calculated using the disability adjusted life year (DALY) model |
| Gkogka et al | 2011 | Risk-based Estimate of Effect of Foodborne Diseases on Public Health, Greece |
| Harris et al | 2019 | Estimating Disability-Adjusted Life Years (DALYs) in Community Cases of Norovirus in England |
| Kristensen et al | 2016 | Burden of four vaccine preventable diseases in older adults |
| Kretzschmar et al | 2012 | New Methodology for Estimating the Burden of Infectious Diseases in Europe |
| Lackner et al | 2019 | The disease burden associated with Campylobacter spp. in Germany, 2014 |
| de Noordhout et al | 2017 | Burden of salmonellosis, campylobacteriosis and listeriosis: a time series analysis, Belgium, 2012 to 2020 |
| Mangen et al | 2013 | The Pathogen- and Incidence-Based DALY Approach: An Appropriated Methodology for Estimating the Burden of Infectious Diseases |
| Mangen et al | 2016 | The burden of Campylobacter-associated disease in six European countries |
| Mastrandrea et al | 2012 | A retrospective study on burden of human echinococcosis based on Hospital Discharge Records from 2001 to 2009 in Sardinia, Italy |
| Pifarré iArolas et al | 2021 | Years of life lost to COVID‑19 in 81 countries |
| Marstrand et al | 2021 | The disease burden of ocular toxoplasmosis in Denmark in 2019: Estimates based on laboratory testing of ocular samples and on publicly available register data |
| Moran et al | 2022 | Estimating the direct Disability-Adjusted Life Years (DALYs) associated with SARS-CoV-2 (COVID-19) in the Republic of Ireland: The first full year |
| Wyper et al | 2021 | Inequalities in population health loss by multiple deprivation: COVID-19 and pre-pandemic all-cause disability-adjusted life years (DALYs) in Scotland |
| Wyper et al | 2022 | Measuring disability-adjusted life years (DALYs) due to COVID-19 in Scotland, 2020 |
| Wyper et al | 2022 | Widening of inequalities in COVID-19 years of life lost from 2020 to 2021: a Scottish Burden of Disease Study |
| Williams et al | 2022 | Years of life lost to COVID-19 in 20 countries |
| Meijerink et al | 2017 | Modelling the burden of hepatitis C infection among people who inject drugs in Norway |
| Müller et al | 2017 | Assessment of the risk of foodborne transmission and burden of hepatitis E in Switzerland |
| Nissen et al | 2017 | The disease burden of congenital toxoplasmosis in Denmark, 2014 |
| Nurchis et al | 2020 | Impact of the burden of COVID-19 in Italy: Results of Disability-Adjusted Life Years (DALYs) and productivity loss |
| Oh et al | 2020 | Years of Life Lost Attributable to COVID-19 in High-incidence Countries |
| Papadopoulos et al | 2019 | The health and economic impact of acute gastroenteritis in Belgium, 2010–2014 |
| Pires et al | 2014 | Burden of Disease of Foodborn Pathogens in Denmark |
| Pires et al | 2019 | Burden of Disease Estimates of Seven Pathogens Commonly Transmitted Through Foods in Denmark, 2017 |
| Plass et al | 2014 | The disease burden of hepatitis B, influenza, measles and salmonellosis in Germany: first results of the Burden of Communicable Diseases in Europe Study |
| Rommel et al | 2021 | The COVID-19 Disease Burden in Germany in 2020-Years of Life Lost to Death and Disease Over the Course of the Pandemic |
| Rypdal et al | 2021 | Estimation of excess mortality and years of life lost to covid-19 in norway and sweden between march and november 2020 |
| Sabbatucci et al | 2019 | Estimated burden of Chlamydia trachomatis female infection and consequent severe pelvic inflammatory disease, Italy, 2005-2016 |
| Šmit & Postma | 2015 | The Burden of Tick-Borne Encephalitis in Disability-Adjusted Life Years (DALYs) for Slovenia |
| Tariq et al | 2011 | Cost of Illness and Disease Burden in The Netherlands Due to Infections with Shiga Toxin–Producing Escherichia coli O157 |
| Toljander et al | 2012 | Public health burden due to infections by verocytotoxin-producing Escherichia coli (VTEC) and Campylobacter spp. as estimated by cost of illness and different approaches to model disability-adjusted life years |
| Torgerson et al | 2008 | Alveolar echinococcosis: from a deadly disease to a well-controlled infection. Relative survival and economic analysis in Switzerland over the last 35 years |
| Torgerson et al | 2010 | The Global Burden of Alveolar Echinococcosis |
| Torgerson et al | 2015 | Global Burden of Leptospirosis: Estimated in Terms of Disability Adjusted Life Years |
| Torgerson et al | 2015 | World Health Organization estimates of the global and regional disease burden of 11 foodborne parasitic diseases, 2010: a data synthesis |
| Rahamat et al | 2006 | State of Infectious Diseases in the Netherlands, 2000 - 2005 |
| Bijkerk et al | 2011 | State of Infectious Diseases in the Netherlands, 2010 |
| Bijkerk et al | 2014 | State of Infectious Diseases in the Netherlands, 2013 |
| Bijkerk et al | 2016 | State of Infectious Diseases in the Netherlands, 2015 |
| de Gier et al | 2016 | State of Infectious Diseases in the Netherlands 2016 |
| de Gier et al | 2017 | State of Infectious Diseases in the Netherlands 2017 |
| de Gier et al | 2019 | State of Infectious Diseases in the Netherlands 2018 |
| Lagerweij et al | 2021 | State of Infectious Diseases in the Netherlands 2019 |
| Klous et al | 2021 | State of Infectious Diseases in the Netherlands 2020 |
| Haagsma et al | 2009 | Disease burden and costs of selected foodborne pathogens in the Netherlands, 2006 |
| Bouwknegt et al | 2013 | Disease burden of food-related pathogens in the Netherlands, 2011 |
| Bouwknegt et al | 2014 | Disease burden of food-related pathogens in the Netherlands, 2012 |
| Mangen et al | 2017 | Disease burden of food-related pathogens in the Netherlands, 2016 |
| Pijnacker et al | 2018 | Disease burden of food-related pathogens in the Netherlands, 2018 |
| Lagerweij et al | 2020 | Disease burden of food-related pathogens in the Netherlands, 2019 |
| de Gier et al | 2018 | Disease burden of psittacosis in the Netherlands |
| de Gier et al | 2019 | Disease burden of neonatal invasive Group B Streptococcus infection in the Netherlands |
| Haagsma et al | 2008 | Disability Adjusted Life Years and minimal disease: application of a preference-based relevance criterion to rank enteric pathogens |
| Haagsma et al | 2010 | Disease burden of post-infectious irritable bowel syndrome in The Netherlands |
| Havelaar et al | 2000 | Health burden in the Netherlands due to infection with thermophilic Campylobacter spp |
| Havelaar et al | 2004 | Disease burden in the Netherlands due to infections with Shiga-toxin producing Escherichia coli O157 |
| Havelaar et al | 2007 | Disease Burden of Congenital Toxoplasmosis |
| Havelaar et al | 2012 | Disease burden of foodborne pathogens in the Netherlands, 2009 |
| Kirk et al | 2015 | World Health Organization Estimates of the Global and Regional Disease Burden of 22 Foodborne Bacterial, Protozoal, and Viral Diseases, 2010: A Data Synthesis |
| Havelaar et al | 2015 | World Health Organization Global Estimates and Regional Comparisons of the Burden of Foodborne Disease in 2010 |
| de Noordhout et al | 2014 | The global burden of listeriosis: a systematic review and meta-analysis |
| Kortbeek et al | 2009 | Congenital toxoplasmosis and DALYs in the Netherlands |
| McDonald et al | 2012 | The impact of demographic change on the estimated future burden of infectious diseases: examples from hepatitis B and seasonal influenza in the Netherlands |
| McDonald et al | 2013 | Effects of an ageing population and the replacement of immune birth cohorts on the burden of hepatitis A in the Netherlands |
| McDonald et al | 2017 | Disease burden of human papillomavirus infection in the Netherlands, 1989–2014: the gap between females and males is diminishing |
| McDonald et al | 2018 | Years of Life Lost Due to Influenza-Attributable Mortality in Older Adults in the Netherlands: A Competing-Risks Approach |
| Monge et al | 2019 | Accounting for long-term manifestations of Cryptosporidium spp infection in burden of disease and cost-of-illness estimations, the Netherlands (2013-2017) |
| van Lier et al | 2019 | Disease burden of varicella versus other vaccine- preventable diseases before introduction of vaccination into the national immunisation programme in the Netherlands |
| Verhoef et al | 2013 | The estimated disease burden of norovirus in The Netherlands |
| Vijgen et al | 2007 | Disease burden and related costs of cryptosporidiosis and giardiasis in the Netherlands |
| van den Wijngaard et al | 2012 | Comparing Pandemic to Seasonal Influenza Mortality: Moderate Impact Overall but High Mortality in Young Children |
| van den Wijngaard et al | 2015 | The burden of Lyme borreliosis expressed in disability-adjusted life years |
| Wielders et al | 2012 | The burden of 2009 pandemic influenza A(H1N1) in the Netherlands |
| Mangen et al | 2004 | Campylobacteriosis and sequelae in the Netherlands |
| Kemmeren et al | 2006 | Priority setting of foodborne pathogens. Disease burden and costs of selected enteric pathogens |
| van Lier & Havelaar | 2007 | Disease burden of infectious diseases in Europe: a pilot study |
| Teirlinck et al | 2016 | Surveillance of influenza and other respiratory infections in the Netherlands: winter 2015/2016 (Annual report) |
| Teirlinck et al | 2017 | Surveillance of influenza and other respiratory infections in the Netherlands: winter 2016/2017 (Annual report) |
| Reukers et al | 2018 | Surveillance of influenza and other respiratory infections in the Netherlands: winter 2017/2018 (Annual report) |
| Reukers et al | 2019 | Surveillance of influenza and other respiratory infections in the Netherlands: winter 2018/2019. (Annual report) |
| Reukers et al | 2020 | Surveillance of influenza and other respiratory infections in the Netherlands: winter 2019/2020 (Annual report) |
| NHS Health Scotland | 2015 | Scottish Burden of Disease Study, 2015: Lower respiratory infections technical overview |
| NHS Health Scotland | 2016 | Scottish Burden of Disease Study, 2016: Lower respiratory infections technical overview |
| Zacher et al | 2019 | Application of a new methodology and R package reveals a high burden of healthcare-associated infections (HAI) in Germany compared to the average in the European Union/European Economic Area, 2011 to 2012 |

**Chapter 4**

| **Author** | **YLL life table** | **YLD approach** | **Disability weights** | **Age-weighting** | **Time-discounting** | |
| --- | --- | --- | --- | --- | --- | --- |
| Becker et al | NA | Incidence-based | GBD | No | No | NA |
| Bermudez-Tamayo et al | GBD standardard model life tables | Prevalence-based | GBD | NR | NR | NA |
| Bordino et al | National | Incidence-based | GBD | No | Yes | With and without a 1.5% time discount rate |
| Brooke et al | National | Incidence-based | GBD | No | No | NA |
| Cassini et al | GBD standardard model life tables | Incidence-based | European DWs | No | No | NA |
| Cassini et al | GBD standardard model life tables | Incidence-based | European DWs | No | No | NA |
| Cassini et al | GBD standardard model life tables | Incidence-based | European DWs | No | No | NA |
| Cassini et al | GBD standardard model life tables | Incidence-based | European DWs | No | No | NA |
| Colzani et al | GBD standardard model life tables | Incidence-based | GBD | No | No | NA |
| Cortés et al | GBD standardard model life tables | Incidence-based | GBD | No | No | NA |
| Cuschieri et al | GBD standardard model life tables | Incidence-based | GBD | No | No | NA |
| Cuschieri & Grech | GBD standardard model life tables | NA | NA | No | No | NA |
| Cuschieri et al | GBD standardard model life tables | NA | NA | No | No | NA |
| Devleesschauwer et al | GBD standardard model life tables | Incidence-based | GBD | No | No | NA |
| Farangel et al | GBD standardard model life tables | Incidence-based | GBD | No | No | NA |
| Fastl et al | GBD standardard model life tables | Incidence-based | GBD | No | No | NA |
| Fan et al | LE data from United Nations | Incidence-based | GBD | Yes | Yes | 3% |
| Gasser et al | GBD standardard model life tables | Incidence-based | European DWs | No | No | NA |
| Garcia-Fulgueiras et al | GBD standardard model life tables | NA | NA | Yes | Yes | 3% |
| Garcia-Fulgueiras et al | GBD standardard model life tables | Prevalence-based | Dutch & Victorian | Yes | Yes | With and without a 3% time discount rate |
| Gaunt et al | National | Incidence-based | GBD | Yes | Yes | 3% |
| Gkogka et al | National | Incidence-based | GBD & DDW | No | No | NA |
| Harris et al | NR | Incidence-based | GBD | NR | NR | NA |
| Kristensen et al | GBD standardard model life tables | Incidence-based | GBD | No | No | NA |
| Kretzschmar et al | GBD standardard model life tables | Incidence-based | GBD | No | No | NA |
| Lackner et al | GBD standardard model life tables | Incidence-based | European DWs | No | No | NA |
| de Noordhout et al | GBD standardard model life tables | Incidence-based | European DWs | No | No | NA |
| Mangen et al | GBD standardard model life tables | Incidence-based | GBD | No | No | NA |
| Mangen et al | GBD standardard model life tables | Incidence-based | GBD | No | Yes | With and without a 3% & 6% time discount rate |
| Mastrandrea et al | NR | Incidence-based | GBD | No | Yes | 3% |
| Pifarré iArolas et al | GBD standardard model life tables | NA | NA | NA | NA | NA |
| Marstrand et al | National | Incidence-based | GBD | NR | NR | NA |
| Moran et al | GBD standardard model life tables | Incidence-based | GBD | No | No | NA |
| Wyper et al | GBD standardard model life tables | Incidence-based | GBD | No | No | NA |
| Wyper et al | GBD standardard model life tables | Incidence-based | GBD | No | No | NA |
| Wyper et al | GBD standardard model life tables | NA | NA | NA | NA | NA |
| Williams et al | GBD standardard model life tables | NA | NA | NA | NA | NA |
| Meijerink et al | NR | Prevalence-based | GBD | No | No | NA |
| Havelaar et al | GBD standardard model life tables | Incidence-based | Dutch DWs | NR | NR | NA |
| Nissen et al | GBD standardard model life tables | Incidence-based | GBD | No | No | NA |
| Nurchis et al | GBD standardard model life tables | Incidence-based | GBD | Yes | No | NA |
| Oh et al | GBD standardard model life tables | NA | NA | No | No | NA |
| Papadopoulos et al | GBD standardard model life tables | Incidence-based | GBD | No | No | NA |
| Pires et al | National | Incidence-based | GBD | No | No | NA |
| Pires et al | GBD standardard model life tables | Incidence-based | GBD | No | No | NA |
| Plass et al | GBD standardard model life tables | Incidence-based | GBD | No | No | NA |
| Rommel et al | National | Incidence-based | GBD & European DW | No | No | NA |
| Rypdal et al | National | NA | NA | No | No | NA |
| Sabbatucci et al | NR | Incidence-based | NR | No | No | NA |
| Šmit & Postma | GBD standardard model life tables | Incidence-based | GBD | No | No | NA |
| Havelaar et al | National | Incidence-based | Dutch DWs | NR | NR | NA |
| Mangen et al | National | Incidence-based | Dutch DWs | No | Yes | With and without a 3% time discount rate |
| Havelaar et al | National | Incidence-based | Dutch DWs | NR | NR | NA |
| Torgerson et al | National | Incidence-based | GBD | Yes | No | NA |
| Torgerson et al | GBD standardard model life tables | Incidence-based | GBD | No | No | NA |
| Torgerson et al | GBD standardard model life tables | Incidence-based | GBD | No | No | NA |
| Rahamat et al | GBD standardard model life tables | Incidence-based | GBD | No | No | NA |
| Bijkerk et al | GBD standardard model life tables | Incidence-based | GBD | No | No | NA |
| Bijkerk et al | GBD standardard model life tables | Incidence-based | GBD | No | No | NA |
| Bijkerk et al | GBD standardard model life tables | Incidence-based | European DWs | No | No | NA |
| de Gier et al | GBD standardard model life tables | Incidence-based | European DWs | No | No | NA |
| de Gier et al | GBD standardard model life tables | Incidence-based | European DWs | No | No | NA |
| de Gier et al | GBD standardard model life tables | Incidence-based | European DWs | No | No | NA |
| Lagerweij et al | GBD standardard model life tables | Incidence-based | European DWs | No | No | NA |
| Klous et al | GBD standardard model life tables | Incidence-based | European DWs | No | No | NA |
| Vijgen et al | National | Incidence-based | Dutch DWs | No | No | NA |
| Torgerson et al | National | Incidence-based | Dutch DWs | Yes | Yes | 3% |
| Bouwknegt et al | National | Incidence-based | GBD | No | No | NA |
| Mangen et al | GBD standardard model life tables | Incidence-based | European DWs | No | No | NA |
| Pijnacker et al | GBD standardard model life tables | Incidence-based | European DWs | No | No | NA |
| Lagerweij et al | GBD standardard model life tables | Incidence-based | European DWs | No | No | NA |
| de Gier et al | GBD standardard model life tables | Incidence-based | European DWs | No | No | NA |
| de Gier et al | GBD standardard model life tables | Incidence-based | European DWs | No | No | NA |
| Haagsma et al | National | Incidence-based | Dutch DWs | No | No | NA |
| Haagsma et al | National | Incidence-based | Dutch DWs | No | Yes | With and without a 3% time discount rate |
| Kortbeek et al | National | Incidence-based | Dutch DWs | No | No | NA |
| Haagsma et al | NA | Incidence-based | Dutch DWs | No | No | NA |
| Tariq et al | National | Incidence-based | Dutch DWs | No | No | NA |
| Toljander et al | National | Incidence-based | Dutch DWs | No | No | NA |
| Kirk et al | NA | Incidence-based | GBD | No | No | NA |
| Havelaar et al | WHO life tables | Incidence-based | GBD | No | No | NA |
| de Noordhout et al | GBD standardard model life tables | Incidence-based | GBD | No | No | NA |
| Havelaar et al | GBD standardard model life tables | Incidence-based | Dutch DWs | No | Yes | With and without a 1.5% time discount rate |
| McDonald et al | National | Incidence-based | GBD | No | No | NA |
| Wielders et al | National | Incidence-based | Dutch DWs | No | No | NA |
| McDonald et al | National | Incidence-based | NR | No | No | NA |
| McDonald et al | National | NA | NA | No | No | NA |
| Monge et al | GBD standardard model life tables | Incidence-based | European DWs | No | Yes | 1.50% |
| van Lier et al | GBD standardard model life tables | Incidence-based | European DWs | No | No | NA |
| Bouwknegt et al | National | Incidence-based | Dutch DWs | No | Yes | With and without a 1.5% time discount rate |
| McDonald et al | National | Incidence-based | Dutch DWs | No | No | NA |
| van den Wijngaard et al | National | NA | NA | No | No | No |
| Verhoef et al | GBD standardard model life tables | Incidence-based | Dutch DWs | No | Yes | With and without a 1.5% time discount rate |
| van den Wijngaard et al | GBD standardard model life tables | Incidence-based | Dutch DWs | No | No | No |
| Müller et al | Swiss national statistics | Incidence-based | Dutch DWs | No | No | NA |
| Kemmeren et al | National | Incidence-based | GBD | No | Yes | With and without a 4% time discount rate |
| van Lier & Havelaar | European LE | Incidence-based | GBD | No | No | NA |
| Teirlinck et al | GBD standardard model life tables | Incidence-based | NR | No | No | NA |
| Teirlinck et al | GBD standardard model life tables | Incidence-based | European DWs | No | No | NA |
| Reukers et al | GBD standardard model life tables | Incidence-based | European DWs | No | No | NA |
| Reukers et al | GBD standardard model life tables | Incidence-based | European DWs | No | No | NA |
| Reukers et al | GBD standardard model life tables | Incidence-based | European DWs | No | No | NA |
| NHS Health Scotland | National | Incidence-based | GBD | No | No | NA |
| NHS Health Scotland | National | Incidence-based | GBD | No | No | NA |
| Zacher et al | National | Incidence-based | European DWs | No | No | NA |

**Chapter 5**

**Periklis Charalampous^1^**, Juanita A Haagsma^1^, Lea S Jakobsen^2^, Vanessa Gorasso^3,4^, Isabel Noguer^5^, Alicia Padron-Monedero^5^, Rodrigo Sarmiento^5,6^, João Vasco Santos^7,8,9^, Scott A McDonald^10^, Dietrich Plass^11^, Grant M A Wyper^12^, Ricardo Assunção^13^, Balázs Ádám^14^, Ala’a AlKerwi^15^, Jalal Arabloo^16^, Ana Lúcia Baltazar^17^, Boris Bikbov^18^, Maria Borrell-Pages^19^, Iris Brus^1^, Genc Burazeri^20^, Serafeim C Chaintoutis^21^, José Chen-Xu^22,23^, Nino Chkhaberidze^24^, Seila Cilovic-Lagarija^25^, Barbara Corso^26^, Sarah Cuschieri^27^, Carlotta Di Bari^4^, Keren Dopelt^28,29^, Mary Economou^30^, Theophilus I Emeto^31,32,33^, Peter Fantke^34^, Florian Fischer^35^, Alberto Freitas^7,8^, Juan Manuel García-González^36^, Federica Gazzelloni^37^, Mika Gissler^38,39,40,41^, Artemis Gkitakou^42^, Hakan Gulmez^43^, Sezgin Gunes^44,45^, Sebastian Haller^46^, Romana Haneef^47^, Cesar A Hincapié^48,49,50^, Paul Hynds^51^, Jane Idavain^52^, Milena Ilic^53^, Irena Ilic^54^, Gaetano Isola^55^, Zubair Kabir^56^, Maria Kamusheva^57^, Pavel Kolkhir^58,59^, Naime Meriç Konar^60^, Polychronis Kostoulas^61^, Mukhtar Kulimbet^62,63^, Carlo La Vecchia^64^, Paolo Lauriola^65^, Miriam Levi^66^‬‬‬‬‬‬‬‬, Marjeta Majer^67^, Enkeleint A Mechili^68,69^, Lorenzo Monasta^70^, Stefania Mondello^71^, Javier Muñoz Laguna^48,49,50^, Evangelia Nena^72^, Edmond SW Ng^73^, Paul Nguewa^74^, Vikram Niranjan^75^, Iskra Alexandra Nola^67^, Rónán O'Caoimh^76,77^, Marija Obradović^78^, Elena Pallari^79^, Mariana Peyroteo^80^, Vera Pinheiro^81,82^, Nurka Pranjic^83^, Miguel Reina Ortiz^84^, Silvia Riva^85^, Cornelia Melinda Adi Santoso^86^, Milena Santric Milicevic^87^, Tugce Schmitt^88^, Niko Speybroeck^89^, Maximilian Sprügel^90^, Paschalis Steiropoulos^91^, Aleksandar Stevanovic^87^, Lau Caspar Thygesen^92^, Fimka Tozija^93^, Brigid Unim^94^, Hilal Bektaş Uysal^95^, Orsolya Varga^96^, Milena Vasic^97,98^, Rafael José Vieira^7,8^, Vahit Yigit^99^, Brecht Devleesschauwer^4,100^, Sara M Pires^2^

^----------------------------------^

^1^Department of Public Health, Erasmus MC University Medical Center, Rotterdam, The Netherlands.

^2^National Food Institute, Technical University of Denmark, Lyngby, Denmark.

^3^Department of Public Health and Primary Care, Ghent University, Ghent, Belgium.

^4^Department of Epidemiology and Public Health, Sciensano, Brussels, Belgium.

^5^National School of Public Health, Carlos III Institute of Health, Madrid, Spain.

^6^Medicine School, University of Applied and Environmental Sciences, Bogota, Colombia.

^7^CINTESIS@RISE - Centre for Health Technology and Services Research, Health Research Network, Faculty of Medicine of the University of Porto, Porto Portugal.

^8^MEDCIDS - Department of Community Medicine, Information and Health Decision Sciences, Faculty of Medicine, University of Porto, Porto, Portugal.

^9^Public Health Unit, Agrupamentos de Centro de Saúde Grande Porto V - Porto Ocidental, Administração Regional de Saúde do Norte, Porto, Portugal.

^10^Centre for Infectious Disease Control, National Institute for Public Health and the Environment (RIVM), Bilthoven, Netherlands.

^11^Department for Exposure Assessment and Environmental Health Indicators, German Environment Agency, Berlin, Germany.

^12^School of Health & Wellbeing, University of Glasgow, Glasgow, United Kingdom.

^13^Food and Nutrition Department, National Institute of Health Dr. Ricardo Jorge, Lisbon, Portugal.

^14^Institute of Public Health, College of Medicine and Health Sciences, United Arab Emirates University, Al Ain, United Arab Emirates.

^15^Directorate of Health, Service Epidemiology and Statistics, Luxembourg, Luxembourg.

^16^Health Management and Economics Research Center, Health Management Research Institute, Iran University of Medical Sciences, Tehran, Iran.

^17^Scientific-Pedagogical Unit of Dietetics and Nutrition, Coimbra Health School, Polytechnic Institute of Coimbra, Coimbra, Portugal.

^18^Dipartimento di Politiche per la Salute, Istituto di Ricerche Farmacologiche Mario Negri IRCCS, Milan, Italy.

^19^Cardiovascular Program ICCC, Institut d'Investigació Biomèdica Sant Pau (IIB-Sant Pau), Barcelona, Spain.

^20^Department of Public Health, Faculty of Medicine, University of Medicine, Tirana, Albania.

^21^Diagnostic Laboratory, School of Veterinary Medicine, Faculty of Health Sciences, Aristotle University of Thessaloniki, Thessaloniki, Greece.

^22^NOVA National School of Public Health, Public Health Research Centre, Universidade NOVA de Lisboa, Lisbon, Portugal.

^23^ Public Health Unit, Primary Health Care Cluster Baixo Mondego, Coimbra, Portugal.

^24^Department of Medical Statistics, National Center for Disease Control and Public Health of Georgia, Georgia.

^25^Institute for Public Health FB&H, Sarajevo, Bosnia and Herzegovina.

^26^Neuroscience Institute, National Research Council, Padova, Italy.

^27^Department of Anatomy, Faculty of Medicine and Surgery, University of Malta, Msida, Malta.

^28^Department of Public Health, Ashkelon Academic College, Ashkelon, Israel.

^29^Department of Health Policy and Management, School of Public Health, Faculty of Health Sciences, Ben-Gurion University of the Negev, Beer Sheva, Israel.

^30^Department of Nursing, School of Health Sciences, Cyprus University of Technology, Limassol, Cyprus.

^31^Public Health & Tropical Medicine, College of Public Health, Medical and Veterinary Sciences, James Cook University, Townsville, Australia.

^32^World Health Organization Collaborating Center for Vector-Borne and Neglected Tropical Diseases, College of Public Health, Medical and Veterinary Sciences, James Cook University, Townsville, Australia.

^33^Australian Institute of Tropical Health and Medicine, James Cook University, Townsville, Australia.

^34^Quantitative Sustainability Assessment, Department of Environmental and Resource Engineering, Technical University of Denmark, Lyngby, Denmark.

^35^Institute of Public Health, Charité - Universitätsmedizin Berlin, Berlin, Germany.

^36^Department of Sociology, Universidad Pablo de Olavide, Seville, Spain.

^37^Institute and Faculty of Actuaries, London, United Kingdom.

^38^Department of Knowledge Brokers, Finnish Institute for Health and Welfare (THL), Helsinki, Finland.

^39^Department of Molecular Medicine and Surgery, Karolinska Institute, Stockholm, Sweden.

^40^Academic Primary Health Care Centre, Stockholm, Sweden.

^41^Research Centre for Child Psychiatry and Invest Research Flagship, University of Turku, Turku, Finland.

^42^Department of Internal Medicine and Epidemiology, Erasmus MC University Medical Center, Rotterdam, The Netherlands.

^43^Department of Family Medicine, Faculty of Medicine, İzmir Democracy University, Izmir, Turkey.

^44^Department of Multidisciplinary Molecular Medicine, Graduate Institute, Ondokuz Mayis University, Samsun, Turkey.

^45^Department of Medical Biology, Medical Faculty, Ondokuz Mayis University, Samsun, Turkey.

^46^Department of Infectious Disease Epidemiology, Robert Koch Institute, Berlin, Germany.

^47^Department of Non-Communicable Diseases and Injuries, Santé Publique France, Saint-Maurice, France.

^48^EBPI-UZWH Musculoskeletal Epidemiology Research Group, University of Zurich and Balgrist University Hospital, Zurich, Switzerland.

^49^Epidemiology, Biostatistics and Prevention Institute (EBPI), University of Zurich, Zurich, Switzerland.

^50^University Spine Centre Zurich (UWZH), Balgrist University Hospital, University of Zurich, Zurich, Switzerland.

^51^Environmental Sustainability and Health Institute, Technological University Dublin, Dublin, Ireland.

^52^Department of Health Statistics, National Institute for Health Development, Tallinn, Estonia.

^53^Department of Epidemiology, Faculty of Medical Sciences, University of Kragujevac, Kragujevac, Serbia.

^54^Faculty of Medicine, University of Belgrade, Belgrade, Serbia.

^55^Department of General Surgery and Surgical-Medical Specialties, School of Dentistry, University of Catania, Catania, Italy.

^56^Public Health & Epidemiology, School of Public Health, University College Cork, Cork, Ireland.

^57^Department of Organization & Economics of Pharmacy, Faculty of Pharmacy, Medical University of Sofia, Sofia, Bulgaria.

^58^Institute of Allergology, Charité - Universitätsmedizin Berlin, corporate member of Freie Universität Berlin, and Humboldt-Universität zu Berlin, Berlin, Germany.

^59^Fraunhofer Institute for Translational Medicine and Pharmacology ITMP, Allergology and Immunology, Berlin, Germany.

^60^Department of Biostatistics and Medical Informatics, Faculty of Medicine, Kirsehir Ahi Evran University, Kirsehir, Turkey.

^61^Laboratory of Epidemiology and Artificial Intelligence, Faculty of Public Health, University of Thessaly, Thessaly, Greece.

^62^Health Research Institute, Al Farabi Kazakh National University, Almaty, Kazakhstan.

^63^Atchabarov Scientific Research Institute of Fundamental Medicine, Asfendiyarov Kazakh National Medical University, Almaty, Kazakhstan.

^64^Department of Clinical Sciences and Community Health, University of Milan, Milan, Italy.

^65^Italian Network of Sentinel Phycians for the Environment (RIMSA), International Society Doctors for the Environment (ISDE), Federazione Nazione Ordine dei Medici (FNOMCeO), Arezzo, Italy.

^66^Epidemiology Unit, Department of Prevention, Local Health Authority Tuscany Centre, Florence, Italy.

^67^Andrija Štampar School of Public Health, School of Medicine, University of Zagreb, Zagreb, Croatia.

^68^Clinic of Social and Family Medicine, School of Medicine, University of Crete, Crete, Greece.

^69^Department of Healthcare, Faculty of Public Health, University of Vlora, Vlora, Albania.

^70^Institute of Maternal, Child Health - IRCCS Burlo Garofolo, Trieste, Italy.

^71^Department of Biomedical and Dental Sciences and Morphofunctional Imaging, University of Messina, Messina, Italy.

^72^Laboratory of Social Medicine, Medical School, Democritus University of Thrace, Alexandroupolis, Greece.

^73^Faculty of Epidemiology and Population Health, London School of Hygiene & Tropical Medicine, London, United Kingdom.

^74^ISTUN Institute of Tropical Health, Department of Microbiology and Parasitology, IdiSNA (Navarra Institute for Health Research), University of Navarra, Pamplona, Spain.

^75^School of Public Health, Physiotherapy and Sport Sciences, University College Dublin, Dublin, Ireland.

^76^Department of Geriatric Medicine, Mercy University Hospital, Grenville Place, Cork City, Ireland.

^77^Department of Gerontology and Rehabilitation, University College Cork, St Finbarr's hospital, Douglas road, Cork City, Ireland.

^78^Department of Preventive and Pediatric Dentistry, Faculty of Medicine, University of Banja Luka, Bosnia and Herzegovina.

^79^Health Innovation Network, Minerva House, Montague Cl, London, United Kingdom.

^80^Comprehensive Health Research Centre, NOVA Medical School, Universidade NOVA de Lisboa, Lisboa, Portugal.

^81^Public Health Unit, Matosinhos Local Health Unit, Matosinhos, Portugal.

^82^CINTESIS - Centre for Health Technology and Services Research, Faculty of Medicine of the University of Porto, Porto Portugal.

^83^Department of Occupational Medicine, School of Medicine, University of Tuzla, Tuzla, Bosnia and Herzegovina.

^84^School of Public and Population Health, Boise State University.

^85^Department of Psychology and Pedagogic Science, St Mary's University, London, United Kingdom.

^86^Faculty of Public Health, University of Debrecen, Debrecen, Hungary.

^87^Institute of Social Medicine, Faculty of Medicine, University of Belgrade, Belgrade, Serbia.

^88^Department of International Health, Care and Public Health Research Institute - CAPHRI, Faculty of Health, Medicine and Life Sciences, Maastricht University, Maastricht, The Netherlands.

^89^Institute of Health and Society (IRSS), Université catholique de Louvain, Brussels, Belgium.

^90^Department of Neurology, Friedrich-Alexander-Universität Erlangen-Nürnberg, Erlangen, Germany.

^91^Department of Respiratory Medicine, Medical School, Democritus University of Thrace, Alexandroupolis, Greece.

^92^National Institute of Public Health, University of Southern Denmark, Denmark.

^93^Faculty of Medicine, Saints Cyril and Methodius University of Skopje, Skopje, North Macedonia.

^94^Department of Cardiovascular, Endocrine-Metabolic Diseases and Aging, Istituto Superiore Di Sanità, Rome, Italy.

^95^Department of Internal Medicine, Adnan Menderes University School of Medicine, Aydin, Turkey.

^96^Department of Public Health and Epidemiology, Faculty of Medicine, University of Debrecen, Debrecen, Hungary.

^97^Faculty of Dentistry Pancevo, University Business Academy in Novi Sad, Pancevo, Serbia.

^98^Institute of Public Health of Serbia Dr Milan Jovanović Batut, Belgrade, Serbia.

^99^Department of Health Management, Suleyman Demirel University, Isparta, Turkey.

^100^Department of Translational Physiology, Infectiology and Public Health, Ghent University, Merelbeke, Belgium.
